# Association of Monocyte Count With Lung Function and Exercise Capacity Among Hospitalized COVID‐19 Survivors: A 2‐Year Cohort Study

**DOI:** 10.1111/irv.13263

**Published:** 2024-03-19

**Authors:** Xiaoying Gu, Lixue Huang, Xia Li, Yuting Zhou, Hui Zhang, Yeming Wang, Dan Cui, Ting Yu, Yimin Wang, Bin Cao

**Affiliations:** ^1^ National Center for Respiratory Medicine Beijing China; ^2^ State Key Laboratory of Respiratory Health and Multimorbidity Beijing China; ^3^ National Clinical Research Center for Respiratory Diseases Beijing China; ^4^ Institute of Respiratory Medicine Chinese Academy of Medical Sciences Beijing China; ^5^ Department of Clinical Research and Data Management, Center of Respiratory Medicine China‐Japan Friendship Hospital Beijing China; ^6^ Department of Respiratory and Critical Care Medicine, Beijing Hospital, National Center of Gerontology, Institute of Geriatric Medicine Chinese Academy of Medical Sciences Beijing China; ^7^ Hubei Provincial Clinical Research Center for Infectious Diseases, Wuhan Research Center for Communicable Disease Diagnosis and Treatment Chinese Academy of Medical Sciences Wuhan China; ^8^ Department of Pulmonary and Critical Care Medicine, Hubei Provincial Clinical Research Center for Infectious Diseases, Wuhan Research Center for Communicable Disease Diagnosis and Treatment Chinese Academy of Medical Sciences Wuhan China; ^9^ Department of Pulmonary and Critical Care Medicine, Center of Respiratory Medicine China‐Japan Friendship Hospital Beijing China; ^10^ Department of Pulmonary and Critical Care Medicine Capital Medical University Beijing China; ^11^ Department of Pulmonary and Critical Care Medicine The 2nd Affiliated Hospital of Harbin Medical University Harbin China; ^12^ Tsinghua University‐Peking University Joint Center for Life Sciences Beijing China

**Keywords:** 6‐min walking distance, healthcare use, long COVID, lung function, monocyte, quality of life

## Abstract

**Background:**

Abnormal changes of monocytes have been observed in acute COVID‐19, whereas associations of monocyte count with long COVID were not sufficiently elucidated.

**Methods:**

A cohort study was conducted among COVID‐19 survivors discharged from hospital. The primary outcomes were core symptoms of long COVID, distance walked in 6 min, and lung function, and the secondary outcomes were health‐related quality of life and healthcare use after discharge. Latent variable mixture modeling was used to classify individuals into groups with similar trajectory of monocyte count from discharge to 2‐year after symptom onset. Multivariable adjusted generalized linear regression models and logistic regression models were used to estimate the associations of monocyte count trajectories and monocyte count at discharge with outcomes.

**Results:**

In total, 1389 study participants were included in this study. Two monocyte count trajectories including high to normal high and normal trajectory were identified. After multivariable adjustment, participants in high to normal high trajectory group had an odds ratio (OR) of 2.52 (95% CI, 1.44–4.42) for smell disorder, 2.27 (1.27–4.04) for 6‐min walking distance less than lower limit of normal range, 2.45 (1.08–5.57) for total lung capacity (TLC) < 80% of predicted, 3.37 (1.16–9.76) for personal care problem, and 1.70 (1.12–2.58) for rehospitalization after discharge at 2‐year follow‐up compared with those in normal trajectory group. Monocyte count at discharge showed similar results, which was associated with smell disorder, TLC < 80% of predicted, diffusion impairment, and rehospitalization.

**Conclusions:**

Monocyte count may serve as an easily accessible marker for long‐term management of people recovering from COVID‐19.

## 1 Introduction

COVID‐19 is now recognized as a heterogeneous disease with a broad spectrum of symptom manifestations and varied disease severity, which is largely determined by variability of the host immune response following SARS‐CoV‐2 infection [1, 2]. Mononuclear phagocyte system (MPS), consisting mainly of monocytes and macrophages, has been reported to cause dysregulated immune response and the hyperinflammatory syndrome during acute COVID‐19 infection [3, 4]. Previous studies about monocyte count with disease severity of COVID‐19 during acute phase were inconsistent. Some studies with limited sample size found no significant differences in circulating monocyte counts among groups with different disease severity [5, 6], whereas other studies found a decrease of monocyte count in the critical patients compared to mild and severe patients [7]. Furthermore, an increase in classical monocyte and a decrease in nonclassical monocyte were found in peripheral blood of COVID‐19 patients, which was considered to be key determinant of severe COVID‐19 [5, 7, 8]. The proportion of inflammatory monocyte‐derived alveolar macrophage (MoAMs) was increased in bronchoalveolar fluid of severe COVID‐19 patients compared with mild COVID‐19 or healthy controls [9]. Hence, abnormal changes of monocytes and MoAMs counts have been observed in acute COVID‐19, but it is unclear whether they can return to normal during the recovery period in patients of varying severity and how long it will take.

In addition to host factors that determine disease severity at acute phase, circulating monocytes may also be related to host factors that can determine long‐term consequences of COVID‐19 [10, 11], which was defined as “long COVID” or “post COVID condition” [12]. The recovery process of multiple diseases including COVID‐19 depends on MPS. Higher circulating monocyte counts were previously reported to be associated with shortened survival and disease progression in patients with fibrotic diseases, including idiopathic pulmonary fibrosis (IPF), systemic sclerosis, myelofibrosis, and hypertrophic cardiomyopathy [13, 14]. However, few follow‐up studies conducted among COVID‐19 survivors have focused on the longitudinal evolution of monocyte counts during convalescence and their association with long‐term outcomes, including respiratory outcomes such as reduced lung function, aerobic capacity and endurance, quality of life, and healthcare use after discharge.

The aims of this study were to assess the relationships of monocyte count trajectories from discharge to 2‐year follow‐up with primary outcomes including core symptoms of long COVID, distance walked in 6 min, and lung function and the secondary outcomes including health‐related quality of life (HRQoL) and healthcare use after discharge at 2 years after symptom onset among hospitalized COVID‐19 survivors. Furthermore, the associations of monocyte count at discharge and highest monocyte count at acute phase with above outcomes were also explored.

## 2 Methods

### 2.1 Study Design and Participants

Participants for the current study were from a cohort study of COVID‐19 survivors discharged from hospital between January 7 and May 29, 2020. Inclusion and exclusion criteria of the cohort study have been described previously [15]. Briefly, all patients with laboratory confirmed COVID‐19 discharged from hospital between January 7 and May 29, 2020, were eligible for participation. Patients were excluded if they died after discharge and before first follow‐up; were living in a nursing or welfare home; had psychotic disorder, dementia, or osteoarthropathy; or were immobile. Data at acute phase were retrieved from electronic medical records (Supporting Information S1: Methods Supplement). Three follow‐up surveys were conducted at 6 months, 1 year, and 2 years after symptom onset.

**FIGURE 1:**
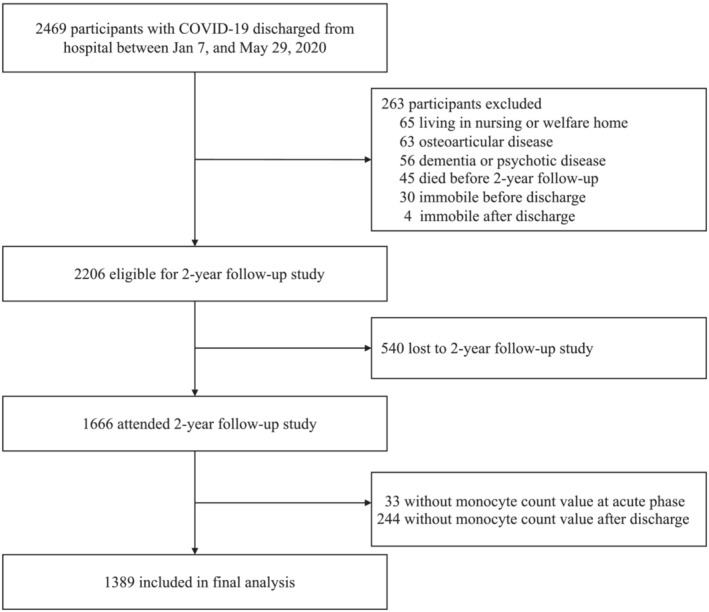
Flow chart of study participants.

Among 2469 participants with COVID‐19, 2206 were eligible to be enrolled in the 2‐year follow‐up study, which was conducted at 2‐year after symptom onset (Figure 1). Of these eligible participants, 1666 (75.5%) completed 2‐year follow‐up survey. After excluding participants without monocyte count value at acute phase (*n* = 33) or without monocyte count value after discharge (*n* = 244), the current analysis was restricted to 1389 participants. The study was approved by the Research Ethics Commission of hospital which enrolled the participants (KY‐2020‐78.01, KY‐2020‐78.03, and KY‐2020‐78.05). Written informed consent was obtained from COVID‐19 survivors who attended the follow‐up visit.

### 2.2 Follow‐Up Assessment

Eligible study participants were invited to attend face‐to‐face follow‐up visits at the outpatient clinic of hospital at 6 months, 1 year, and 2 years after symptom onset. The 6‐month, 1‐year, and 2‐year follow‐up visits were conducted from June 16 to September 3, 2020, from December 16, 2020, to February 7, 2021, and from November 16, 2021, to January 10, 2022, respectively. A telephone survey was available for COVID‐19 survivors at the 2‐year follow‐up visit as an alternative to the face‐to‐face interview, which was conducted by trained clinicians using the same questionnaires. The detailed 6‐month 1‐year, and 2‐year follow‐up procedures have been described previously [15, 16, 17]. Briefly, at each visit, they underwent a detailed interview, a physical examination, and a 6‐min walking distance (6MWD) test, and completed a series of questionnaires, including a self‐reported symptom questionnaire, the modified British Medical Research Council (mMRC) dyspnea scale, the EQ‐5D‐5L questionnaire to assess HRQoL, the EuroQol Visual Analogue Scale (EQ‐VAS; scores range from 0 to 100, with a higher score indicating a better health status), and so forth. Additionally, at the 1‐year and 2‐year visits, healthcare use after discharge was also collected by a questionnaire. Venous blood samples were drawn for the measurement of monocyte count and other laboratory indicators. A stratified disproportional random sampling procedure according to severity scale was used to select patients to receive pulmonary function tests (PFTs) at 6‐month follow‐up visit (Supporting Information S1: Methods Supplement).

### 2.3 Outcomes

The primary outcomes were core symptoms more specifically related to long COVID (fatigue or muscle weakness, smell disorder, taste disorder, and dyspnea defined as mMRC ≥ 1) [18, 19], distance walked in 6 min, and lung function (forced expiratory volume in 1 s [FEV_1_] < 80% of predicted, forced vital capacity [FVC] < 80% of predicted, total lung capacity (TLC) < 80% of predicted, and diffusion capacity for carbon monoxide [DLCO] < 80% of predicted). The definition of fatigue or muscle weakness, smell disorder, and taste disorder was self‐reported newly occurring or worse symptoms post COVID‐19. The secondary outcomes were HRQoL (pain or discomfort, anxiety or depression, mobility, usual activity, and personal care) and healthcare use after discharge (outpatients' clinic visit, rehospitalization, and emergency department visit).

### 2.4 Statistical Analysis

To identify different groups of study participants that share similar underlying trajectory of monocyte count from discharge to 2‐year after symptom onset, latent variable mixture modeling was used to classify individuals into groups with similar patterns. The last monocyte count measured during hospitalization was defined as monocyte count at discharge. The median duration from measurement of last monocyte count during hospitalization to discharge was 4 (IQR 3–7) days. We fitted trajectory models and started with a 1‐trajectory model in cubic form. The number of trajectories was determined by taking both the Bayesian information criterion for each set of trajectories and clinical implication into consideration. Finally, two trajectories with one in cubic form and the other in quadratic form were identified.

Demographic and clinical characteristics, clinical outcomes, and healthcare use after discharge of study participants were presented as median (IQR) for continuous variables and expressed as absolute values along with percentages for categorical variables. The comparisons of demographic and clinical characteristics, clinical outcomes, and healthcare use after discharge across different monocyte count trajectories were performed with χ^2^ test, Fisher's exact, or Kruskal–Wallis test where appropriate.

Study participants were also categorized into three groups according to monocyte count at discharge (<0.40 × 10^9^/L, 0.40–<0.60 × 10^9^/L, and ≥0.60 × 10^9^/L) and highest monocyte count at acute phase (<0.40 × 10^9^/L, 0.40–<0.60 × 10^9^/L, and ≥0.60 × 10^9^/L), respectively. The comparisons of demographic and clinical characteristics, clinical outcomes, and healthcare use after discharge across these groups were performed with χ^2^ test, Fisher's exact, or Kruskal–Wallis test where appropriate.

The associations of monocyte count trajectories from discharge to 2‐year follow‐up with distance walked in 6 min and percentage of predicted distance walked in 6 min were assessed with multivariable adjusted generalized linear regression model. Multivariable adjusted logistic regression models were used to explore association of monocyte count trajectories with other outcomes, including core symptoms (fatigue or muscle weakness, smell disorder, taste disorder, and dyspnea), distance walked in 6 min less than lower limit of normal range (LLN), lung function (FEV_1_ < 80% of predicted, FVC < 80% of predicted, TLC < 80% of predicted, and DLCO<80% of predicted), HRQoL (pain or discomfort, anxiety or depression, mobility, usual activity, and personal care), and healthcare use after discharge (outpatients clinic visit, rehospitalization, and emergency department visit). Age, sex, cigarette smoking (never‐smoker, current smoker, and former smoker), education (college or higher vs. high school or lower), body mass index (BMI), comorbidity (hypertension, diabetes, cardiovascular diseases, cerebrovascular diseases, malignant tumor, chronic obstructive pulmonary disease, and chronic kidney disease), disease severity (scale 3, scale 4, and scale 5–6), and corticosteroids use during hospitalization were adjusted in these models.

The associations of monocyte count at discharge and highest monocyte count at acute phase with continuous and categorical outcomes were assessed with generalized linear regression models and logistic regression models, respectively. The covariables adjusted were the same as that adjusted in the modes for exploring association between monocyte count trajectories from discharge to 2‐year follow‐up and outcomes.

All significance tests were two‐sided, and a *p* value less than 0.05 was considered statistically significant. All statistical analyses were done with SAS, version 9.4 (SAS Institute Inc, Cary, NC).

## 3 Results

### 3.1 Monocyte Count Trajectories From Discharge to 2‐Year Follow‐Up

**FIGURE 2:**
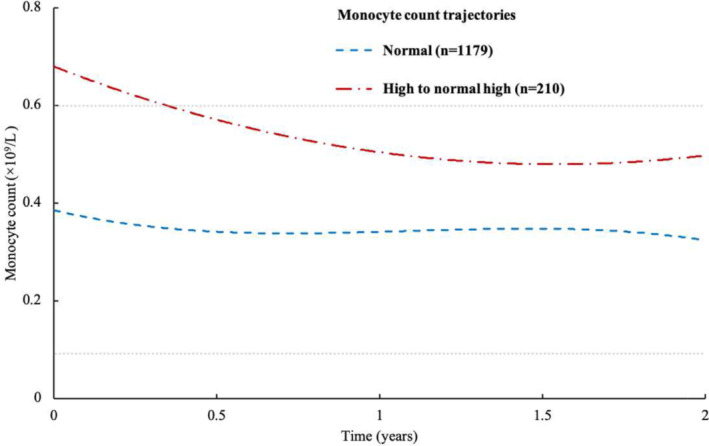
Trajectories of monocyte count from discharge to 2 years after symptom onset among hospitalized COVID‐19 survivors.

As shown in Figure 2, two monocyte count trajectories from discharge to 2‐year after symptom onset were identified. The sensitivity analysis after excluding participants with last monocyte count during hospitalization measured more than 7 days before discharge showed similar monocyte count trajectories from discharge to 2‐year after symptom onset (Figure S1; Supporting Information S1: Results Supplement).

### 3.2 Baseline Characteristics of Study Participants

The demographic and clinical characteristics of 1389 study participants included in this study are presented in Table 1. The median age of study participants was 57.0 (IQR 48.0–65.0) years, and 53% of them was male. Compared with study participants with normal monocyte count trajectory, those with high to normal high trajectory were more likely to be male and smokers, have hypertension, and receive corticosteroids and lopinavir‐ritonavir during hospitalization. The study participants were not vaccinated at 6‐month and 1‐year follow‐up, and 81% of them was vaccinated at 2‐year follow‐up. The proportion of vaccination was slightly higher among participants in normal trajectory group. Furthermore, BMI was higher, and length of intensive care unit (ICU) stay was longer among participants with high to normal high monocyte trajectory compared to those with normal monocyte trajectory.

**TABLE 1:** Baseline characteristics of hospitalized COVID‐19 survivors according to monocyte count trajectories.

	Total (*n* = 1389)	Monocyte count trajectories		*p* value
		Normal (*n* = 1179)	High to normal high (*n* = 210)	
Age, years	57.0 (48.0–65.0)	57.0 (47.0–65.0)	59.0 (49.0–67.0)	0.10
Sex				<0.0001
Male	743 (53%)	580 (49%)	163 (78%)	
Female	646 (47%)	599 (51%)	47 (22%)	
Education				0.14
College or higher	404/1371 (29%)	334/1164 (29%)	70/207 (34%)	
Middle school or lower	967/1371 (71%)	830/1164 (71%)	137/207 (66%)	
Cigarette smoking				<0.0001
Never‐smoker	1151/1388 (83%)	1013/1178 (86%)	138 (66%)	
Current smoker	103/1388 (7%)	68/1178 (6%)	35 (17%)	
Former smoker	134/1388 (10%)	97/1178 (8%)	37 (18%)	
BMI, kg/m^2^	25.0 (22.9–27.3)	24.8 (22.6–27.1)	26.0 (24.2–28.0)	<0.0001
Comorbidity				
Hypertension	500/1388 (36%)	392/1178 (33%)	108 (51%)	<0.0001
Diabetes	200/1388 (14%)	165/1178 (14%)	35 (17%)	0.31
Coronary heart diseases	130/1387 (9%)	107/1178 (9%)	23/209 (11%)	0.38
Cerebrovascular diseases	79/1388 (6%)	65/1178 (6%)	14 (7%)	0.51
Chronic kidney disease	62 (4%)	52 (4%)	10 (5%)	0.82
Malignancy	37 (3%)	30 (3%)	7 (3%)	0.51
COPD	20 (1%)	18 (2%)	2 (1%)	0.50
Highest seven‐category scale during hospital stay				0.21
3: Not requiring supplemental oxygen	341 (25%)	297 (25%)	44 (21%)	
4: Requiring supplemental oxygen	941 (68%)	796 (68%)	145 (69%)	
5: Requiring HFNC or non‐IMV, or both	99 (7%)	81 (7%)	18 (9%)	
6: Requiring ECMO or IMV, or both	8 (1%)	5 (0%)	3 (1%)	
Treatment received during hospital stay				
Corticosteroids	332/1385 (24%)	262/1176 (22%)	70/209 (33%)	0.0005
Antivirals	746/1364 (55%)	623/1156 (54%)	123/208 (59%)	0.16
Lopinavir‐ritonavir	189/1385 (14%)	150/1176 (13%)	39/209 (19%)	0.022
Arbidol	667/1385 (48%)	564/1176 (48%)	103/209 (49%)	0.72
Chloroquine phosphate	4/1385 (0%)	4/1176 (0%)	0/209 (0%)	1.00
Hydroxychloroquine	1/1385 (0%)	0/1176 (0%)	1/209 (0%)	0.15
Antibiotics	1070/1385 (77%)	905/1176 (77%)	165/209 (79%)	0.53
Thymosin	221/1385 (16%)	189/1176 (16%)	32/209 (15%)	0.78
Intravenous immunoglobulin	275/1385 (20%)	229/1176 (19%)	46/209 (22%)	0.40
Length of hospital stay, days	14.0 (10.0–20.0)	14.0 (10.0–20.0)	14.0 (11.0–21.0)	0.17
ICU admission	63 (5%)	52 (4%)	11 (5%)	0.60
Length of ICU stay, days	14.0 (7.0–28.0)	12.0 (6.0–21.5)	30.0 (13.0–55.0)	0.0088
Vaccination status^a^				0.040
Unvaccinated	259/1382 (19%)	212/1172 (18%)	47/210 (22%)	
One dose	361/1382 (26%)	305/1172 (26%)	56/210 (27%)	
Two doses	639/1382 (46%)	558/1172 (48%)	81/210 (39%)	
Three and more doses	123/1382 (9%)	97/1172 (8%)	26/210 (12%)	
Time from symptom onset to 2‐year follow‐up, days	687.0 (676.0–700.0)	688.0 (676.0–700.0)	685.0 (675.0–698.0)	0.07

*Note:* Data are *n* (%), *n*/*N* (%), or median (IQR). The differing denominators used indicate missing data.

Abbreviations: COPD = chronic obstructive pulmonary disease; ECMO = extracorporeal membrane oxygenation; HFNC = high‐flow nasal cannula for oxygen therapy; ICU = intensive care unit; IMV = invasive mechanical ventilation; NA = not applicable.

^a^ Vaccination status at 2‐year follow‐up visit. COVID‐19 survivors in this study were not vaccinated at 6‐month and 1‐year follow‐up.

The comparison of characteristics among participants classified by monocyte count at discharge and by highest monocyte count at acute phase both showed similar patter as the comparison among participants in different trajectories (Table S1‐2; Supporting Information S1: Results Supplement).

### 3.3 Clinical Outcomes and Healthcare Use of Study Participants

Table 2 shows the clinical outcomes and healthcare use of hospitalized COVID‐19 survivors according to monocyte count trajectory from discharge to 2‐year follow‐up. The proportions of study participants with smell disorder, 6‐min walking distance less than LLN, rehospitalization after discharge, and decreased TLC (<80% of predicted value) were higher among participants with high to normal high monocyte count trajectory compared to those with normal trajectory. Although the proportion of DLCO <80% of predicted was numerically higher among participants in the high to normal high trajectory group than those in the normal trajectory group (49% vs 43%), but the difference was not statistically significant (*p* = 0.51). Compared with participants with normal monocyte trajectory, EQ‐VAS score was lower, whereas FEV_1_ was higher among those with high to normal high monocyte trajectory.

**TABLE 2:** Clinical outcomes and healthcare use of hospitalized COVID‐19 survivors at 2‐year follow‐up visits according to monocyte count trajectories.

	Total (*n* = 1389)	Monocyte count trajectories		*p* value
		Normal (*n* = 1179)	High to normal high (*n* = 210)	
Core symptom^a^				
Fatigue or muscle weakness	413/1386 (30%)	352/1176 (30%)	61/210 (29%)	0.80
Smell disorder	72/1386 (5%)	50/1176 (4%)	22/210 (10%)	0.0002
Taste disorder	44/1386 (3%)	38/1176 (3%)	6/210 (3%)	0.78
Dyspnea (mMRC ≥ 1)	202/1387 (15%)	168/1177 (14%)	34/210 (16%)	0.47
Distance walked in 6 min, m	510.0 (458.0–561.5)	510.0 (458.0–558.0)	519.0 (460.0–570.0)	0.49
Percentage of predicted value^b^	94.0 (84.6–104.0)	93.9 (85.0–103.7)	94.3 (83.5–106.8)	0.70
Less than LLN^c^	88/1069 (8%)	66/896 (7%)	22/173 (13%)	0.019
EQ‐5D‐5L questionnaire				
Pain or discomfort	325/1387 (23%)	270/1177 (23%)	55 (26%)	0.31
Anxiety or depression	185/1387 (13%)	163/1177 (14%)	22 (10%)	0.19
Mobility problem	50/1387 (4%)	38/1177 (3%)	12 (6%)	0.08
Usual activity problem	43/1387 (3%)	32/1177 (3%)	11 (5%)	0.05
Personal care problem	19/1387 (1%)	13/1177 (1%)	6 (3%)	0.07
EuroQol VAS score	80.0 (70.0–90.0)	80.0 (70.0–90.0)	80.0 (70.0–90.0)	0.018
Healthcare use after discharge				
Outpatient clinic visit	274/1384 (20%)	231/1175 (20%)	43/209 (21%)	0.76
Rehospitalization	187/1384 (14%)	147/1175 (13%)	40/209 (19%)	0.0098
Emergency department visit	9/1384 (1%)	7/1175 (1%)	2/209 (1%)	0.57
Lung function				
FEV_1_, L	2.9 (2.4–3.4)	2.8 (2.3–3.4)	3.2 (2.6–3.6)	0.038
FVC, L	3.7 (3.0–4.3)	3.6 (3.0–4.3)	4.0 (3.4–4.4)	0.06
TLC, L	5.0 (4.4–5.7)	5.0 (4.3–5.6)	5.3 (4.7–5.9)	0.11
DLCO, mmol/min/kPa	6.8 (5.8–8.0)	6.7 (5.7–8.0)	6.8 (6.3–8.2)	0.39
FEV_1_ < 80%, % of predicted	13/260 (5%)	11/217 (5%)	2/43 (5%)	0.91
FVC < 80%, % of predicted	9/260 (3%)	9/217 (4%)	0/43 (0%)	0.07
TLC < 80%, % of predicted	56/259 (22%)	39/216 (18%)	17/43 (40%)	0.0018
DLCO < 80%, % of predicted	115/260 (44%)	94/217 (43%)	21/43 (49%)	0.51

*Note:* Data are median (IQR), *n* (%), or *n*/*N* (%). The differing denominators used indicate missing data.

Abbreviations: DLCO = diffusion capacity for carbon monoxide; EQ‐5D‐5L = EuroQol five‐dimension five‐level questionnaire; EQ‐VAS = EuroQol Visual Analogue Scale; FEV_1_ = forced expiratory volume in 1 s; FVC = forced vital capacity; LLN = lower limit of normal range; mMRC = modified British Medical Research Council; TLC = total lung capacity.

^a^ Core symptoms were identified as symptoms more specifically related to long COVID according to findings of Mizrahi and Ballering [20, 21].

^b^ Predicted values were calculated according to the method of Enright and Sherrill.

^c^ The lower limit of the normal range was calculated by subtracting 153 m from the predicted value for men or by subtracting 139 m for women.

The clinical outcomes and healthcare use of hospitalized COVID‐19 survivors according to monocyte count at discharge and highest monocyte count during hospitalization were shown in Tables S3 and S4, respectively (Supporting Information S1: Results Supplement).

### 3.4 Association of Monocyte Count Trajectories With Clinical Outcomes and Healthcare Use

**FIGURE 3:**
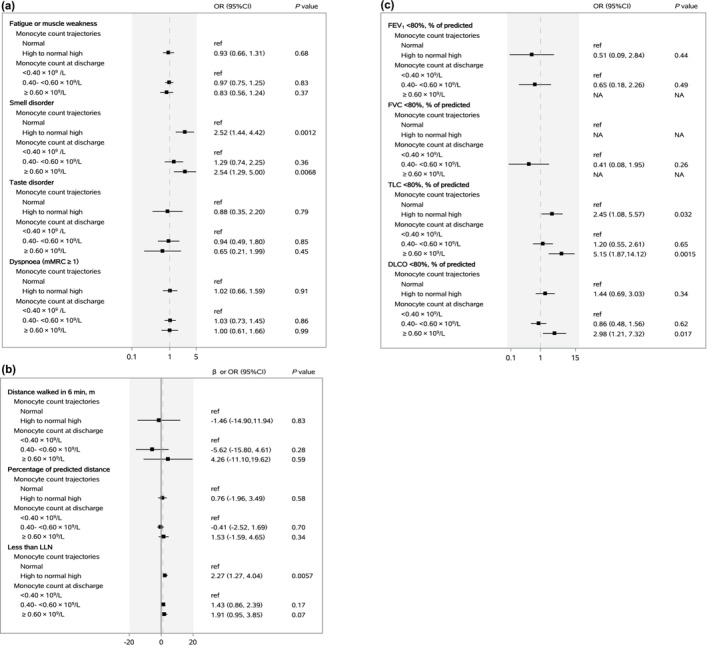
Association of monocyte count trajectories and monocyte count at discharge with core symptoms (a), distance walked in 6 min (b) and lung function (c) at 2‐year follow‐up.

**FIGURE 4:**
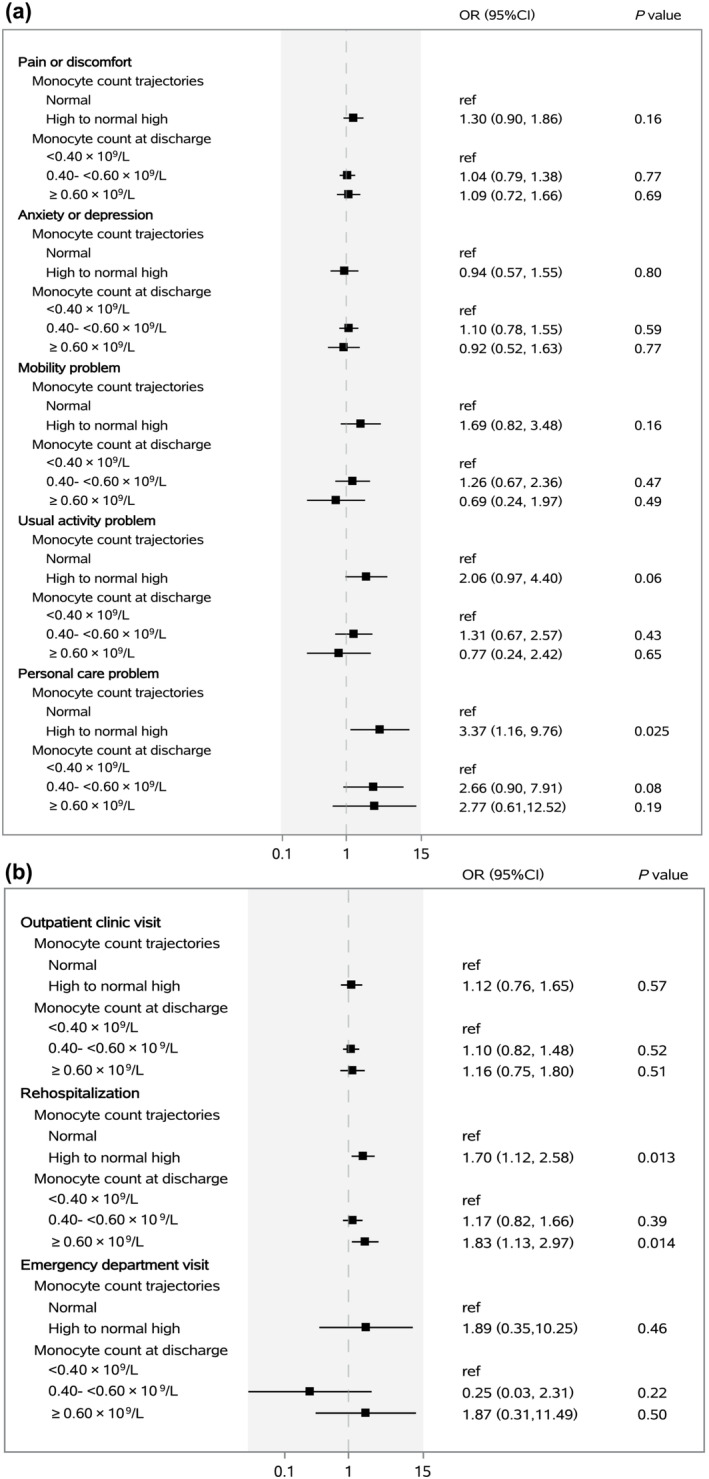
Association of monocyte count trajectories and monocyte count at discharge with health‐related quality of life (a) and healthcare use after discharge (b) at 2‐year follow‐up.

The associations of monocyte count trajectories from discharge to 2‐year after symptom onset with core symptoms, distance walked in 6 min, and lung function at 2‐year follow‐up are shown in Figure 3. Compared with participants in normal trajectory group, the multivariable adjusted odds ratios (ORs) (95% CIs) were 2.52 (1.44–4.42) for smell disorder, 2.27 (1.27–4.04) for 6‐min walking distance less than LLN, and 2.45 (1.08–5.57) for TLC < 80% of predicted value among participants in high to normal high trajectory group. The associations of monocyte count trajectories with HRQoL and healthcare use after discharge at 2‐year follow‐up are shown in Figure 4. After multivariable adjustment, participants in high to normal high trajectory group had an OR of 3.37 (95% CI, 1.16–9.76) for personal care problem and 1.70 (1.12–2.58) for rehospitalization after discharge at 2‐year follow‐up compared with those in normal trajectory group. The associations remained stable with further adjustment for vaccination status at 2‐year follow‐up (Table S5).

The sensitivity analyses with exclusion of participants with last monocyte count during hospitalization measured more than 7 days before discharge did not change the statistically significant association of monocyte count trajectory with smell disorder, 6‐min walking distance less than LLN, and personal care problem (Figures S2 and S3). The associations of monocyte count trajectory with mobility problem and usual activity problem became statistically significant.

### 3.5 Association of Monocyte Count at Discharge With Clinical Outcomes and Healthcare Use

The associations of monocyte count at discharge with clinical outcomes and healthcare use at 2‐year follow‐up are shown in Figures 3 and 4. The multivariable adjusted ORs (95% CIs) were 5.15 (1.87–14.12) for TLC < 80% of predicted and 2.98 (1.21–7.32) for DLCO <80% of predicted in participants with monocyte count ≥0.60 × 10^9^/L at discharge compared with participants with monocyte count <0.40 × 10^9^/L at discharge. Compared with participants with monocyte count <0.40 × 10^9^/L at discharge, the multivariable adjusted ORs (95% CIs) were 1.29 (0.74–2.25) and 2.54 (1.29–5.00) for smell disorder and 1.17 (0.82–1.66) and 1.83 (1.13–2.97) for rehospitalization after discharge in participants with monocyte count 0.40–<0.60 × 10^9^/L and ≥0.60 × 10^9^/L at discharge, respectively.

The sensitivity analyses did not substantially change the association between monocyte count at discharge and clinical outcomes and healthcare use, except that the association with rehospitalization after discharge was not statistically significant (Figures S2 and S3).

## 4 Discussion

**FIGURE 5:**
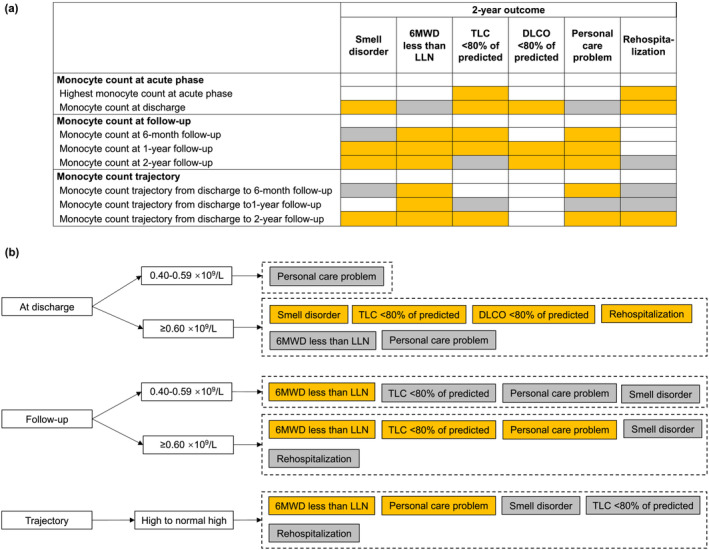
Summary for associations of monocyte count at different time points with outcomes at 2‐year follow‐up.

In the follow‐up study of hospitalized COVID‐19 survivors, we found survivors with high to normal high monocyte count trajectory from discharge to 2‐year after symptom onset had a higher 2‐year odds of smell disorder, poor exercise capacity, decreased TLC, poor HRQoL mainly in the domain of personal care, and all‐cause rehospitalization after discharge compared with those with normal trajectory. Hospitalized COVID‐19 survivors with monocyte count of ≥0.60 × 10^9^/L at discharge had higher 2‐year odds of smell disorder, decreased TLC, diffusion impairment, and all‐cause rehospitalization after discharge versus those with monocyte count of <0.40 × 10^9^/L. To better elucidate the association of monocyte count with long‐term outcomes among COVID‐19 survivors, we further analyzed and summarized associations of monocyte count at all time points available in our study with outcomes at 2‐year follow‐up (Figure 5). The dynamic associations provided evidence for clinicians to make corresponding diagnosis and treatment decisions according to different monocyte level and prognosis status at different periods.

To our knowledge, this is the first study to describe the trajectory of monocyte count up to 2 years after SARS‐CoV‐2 infection. The levels of lymphocyte, neutrophil, and eosinophils at different follow‐up time points were slightly higher among high to normal high trajectory group than that among normal trajectory group (Table S6). The persistent higher level of these peripheral blood markers may indicate a chronic inflammation status. Consistent with our findings, previous studies also found that abnormal peripheral blood monocyte count during acute phase of COVID‐19 had returned to normal level during the convalescence [22, 23], but when they will return to a baseline status before SARS‐CoV‐2 infection was unclear. Elevated monocytes after acute SARS‐CoV‐2 infection were recruited to the airways to differentiate into monocyte‐derived alveolar macrophage, which has been reported to be associated with pulmonary fibrosis by stimulating and forming reciprocal circuits with fibroblasts [24, 25, 26]. In patients with IPF, elevated monocyte count was associated with increased risks of IPF progression, accelerated decline in lung function, hospitalization, and mortality, which indicated monocyte count may serve as a simple and inexpensive prognostic biomarker [13, 14, 20, 27]. However, the role of monocyte count played in the long‐term recovery process of COVID‐19 patients was not clearly delineated. Better understanding the association of post‐acute monocyte count recovery trajectories rather than just focusing monocyte count at acute phase would be more enlightening for long‐term management of people recovering from COVID‐19.

Long COVID has become a global concern, and its underlying pathogenesis was still unclear. One possible reason may be due to the chronic inflammation caused by persistent SARS‐CoV‐2 virus or viral antigens and RNA in multiple tissues [2, 21]. Pathogen‐associated molecular patterns caused by persistent SARS‐CoV‐2 reservoir or remnants could engage various host pattern recognition receptors to trigger innate immune activation [21]. Recently, SARS‐CoV‐2 antigen and RNA were found in tissue macrophages of recovered patients around 1 year after symptom onset [28, 29]. This may have some influence on the stable state of peripheral circulation of monocytes. The current finding of association of monocyte count trajectory from discharge to 2‐year follow‐up, monocyte count at discharge, and highest monocyte count at acute phase and worse outcomes (results for highest monocyte count at acute phase shown in Figures S4 and S5) in COVID‐19 survivors provided us a new insight into the use of monocyte count for identification of COVID‐19 survivors at risk of poor long‐term prognosis. Evaluation of monocyte count and its recovery trajectory, as an easily accessible resource, provides the first indicator of long‐term outcome in the absence of functional information among people recovering from COVID‐19.

Our study has several limitations. First, we mainly focused on monocyte counts without evaluating monocyte function; thus, further studies are needed to explore association of monocyte subset and monocyte activation markers with long‐term outcomes. Second, this is a single‐center study with enrollment of COVID‐19 survivors who discharged at the early stage of pandemic, which may limit the generalizability of study findings. Finally, information bias resulted from recalling information such as smoking and self‐reported comorbidity cannot be excluded, even though misclassification of these variables was more likely to be non‐differential in our cohort study.

The cohort study with 2‐year follow‐up duration after symptom onset among people recovering from COVID‐19 provided evidence for the association of both monocyte count trajectory from discharge to follow‐up and elevated monocyte counts at discharge with increased risk of worse physical or functional outcomes among COVID‐19 survivors. The findings provided clues for monocyte count as an easily accessible marker for long‐term management of people recovering from COVID‐19. Future studies with longer follow‐up duration and larger sample size are needed to more comprehensively understand the role of monocyte served for pathophysiology of long COVID, with focusing on not only monocyte count but also its function.

## Author Contributions

**Xiaoying Gu:** Formal analysis; Investigation; Writing – original draft. **Lixue Huang:** Investigation; Writing – original draft. **Xia Li:** Investigation. **Yuting Zhou:** Investigation. **Hui Zhang:** Investigation. **Yeming Wang:** Investigation. **Dan Cui:** Investigation. **Ting Yu:** Investigation. **Yimin Wang:** Investigation; Writing – review and editing. **Bin Cao:** Funding acquisition; Supervision; Writing – review and editing.

## Ethics Statement

The study was approved by the Research Ethics Commission of hospital which enrolled the participants (KY‐2020‐78.01, KY‐2020‐78.03, and KY‐2020‐78.05).

## Consent

Written informed consent was obtained from COVID‐19 survivors who attended the follow‐up visit.

## Conflicts of Interest

The authors declare no conflicts of interest.

## Supporting information


**Table S1.** Baseline characteristics of hospitalized COVID‐19 survivors according to monocyte count at discharge.
**Table S2.** Baseline characteristics of hospitalized COVID‐19 survivors according to highest monocyte count at acute phase.
**Table S3.** Clinical outcomes and healthcare use of hospitalized COVID‐19 survivors at 2‐year follow‐up visits according to monocyte count at discharge.
**Table S4.** Clinical outcomes and healthcare use of hospitalized COVID‐19 survivors at 2‐year follow‐up visits according to highest monocyte count at acute phase.
**Table S5.** Association of monocyte count trajectories with outcomes with further adjustment for vaccination status at 2‐year follow‐up.
**Table S6.** Laboratory tests of hospitalized COVID‐19 survivors according to monocyte count trajectories.
**Figure S1.** Sensitivity analysis for trajectories of monocyte count from discharge to 2 years after symptom onset among hospitalized COVID‐19 survivors.
**Figure S2.** Sensitivity analysis for association of monocyte count trajectories and monocyte count at discharge with core symptom (a), distance walked in 6 min (b) and lung function (c) at 2‐year follow‐up.
**Figure S3.** Sensitivity analysis for association of monocyte count trajectories and monocyte count at discharge with health‐related quality of life (a) and healthcare use after discharge (b) at 2‐year follow‐up.
**Figure S4.** Association of highest monocyte count at acute phase with core symptom (a), distance walked in 6 min (b) and lung function (c) at 2‐year follow‐up.
**Figure S5.** Association of highest monocyte count at acute phase with health‐related quality of life (a) and healthcare use after discharge (b) at 2‐year follow‐up.

## Data Availability

Restrictions apply to the availability of these data and so are not publicly available. However, data are available from the authors upon reasonable request and with the permission of the institution.
