# Supplementary material for: Association of Monocyte Count With Lung Function and Exercise Capacity Among Hospitalized COVID‐19 Survivors: A 2‐Year Cohort Study
Source: Influenza Other Respir Viruses. 2024 Mar 19;18(3):e13263. doi: 10.1111/irv.13263 (PMC10950557; doi:10.1111/irv.13263)

**Supplementary Appendix**

**Association of monocyte count with lung function and exercise capacity among hospitalized COVID-19 survivors: a 2-year cohort study**

Table of contents

Methods Supplement 3

Results Supplement 4

Table S1. Baseline characteristics of hospitalized COVID-19 survivors according to monocyte count at discharge 6

Table S2. Baseline characteristics of hospitalized COVID-19 survivors according to highest monocyte count at acute phase 8

Table S3. Clinical outcomes and healthcare use of hospitalized COVID-19 survivors at 2-year follow-up visits according to monocyte count at discharge 10

Table S4. Clinical outcomes and healthcare use of hospitalized COVID-19 survivors at 2-year follow-up visits according to highest monocyte count at acute phase 12

Table S5. Association of monocyte count trajectories with outcomes with further adjustment for vaccination status at 2-year follow-up 14

Table S6. Laboratory tests of hospitalized COVID-19 survivors according to monocyte count trajectories 16

Figure S1. Sensitivity analysis for trajectories of monocyte count from discharge to 2 years after symptom onset among hospitalized COVID-19 survivors 17

Figure S2. Sensitivity analysis for association of monocyte count trajectories and monocyte count at discharge with core symptom (a), distance walked in 6 min (b) and lung function (c) at 2-year follow-up 18

Figure S3. Sensitivity analysis for association of monocyte count trajectories and monocyte count at discharge with health-related quality of life (a) and healthcare use after discharge (b) at 2-year follow-up 20

Figure S4. Association of highest monocyte count at acute phase with core symptom (a), distance walked in 6 min (b) and lung function (c) at 2-year follow-up 21

Figure S5. Association of highest monocyte count at acute phase with health-related quality of life (a) and healthcare use after discharge (b) at 2-year follow-up 23

# Methods Supplement

**Data collection at acute phase**

Acute phase was defined as the time between symptom onset and hospital discharge. Data at acute phase was retrieved from electronic medical records, including demographic characteristics (age, sex, education, and cigarette smoking); clinical characteristics (self-reported comorbidities and symptom onset time); laboratory test results (monocyte count); disease severity characterized by highest seven-category scale during hospital stay (3, hospitalized, not requiring supplemental oxygen; 4, hospitalized, requiring supplemental oxygen; 5, hospitalized, requiring high-flow nasal cannula (HFNC), noninvasive mechanical ventilation (NIV), or both; 6, hospitalized, requiring extracorporeal membrane oxygenation (ECMO), invasive mechanical ventilation (IMV), or both); and treatment (corticosteroids, antivirals including lopinavir–ritonavir, arbidol, chloroquine phosphate, and hydroxychloroquine, antibiotics, thymosin, and intravenous immunoglobulin).

**Follow-up assessment**

In light of the emergency state of hospital at the early stage of COVID-19 pandemic, demographic information and self-reported comorbidity collected at admission may not be that accurate. To confirm demographic information and self-reported comorbidity collected at baseline, a standard questionnaire was designed and administered to obtain information including age, gender, education, cigarette smoking, alcohol consumption, personal medical history, and family history face-to-face by trained staff at 1-year and 2-year follow-up visit. All data collected was checked for completeness by the staff who collected information unless study participants were unwilling to provide the information. The data was finally recoded to reflect baseline characteristics of study participants according to time variables collected for some variables such as comorbidity and admission date.

Patients requiring HFNC, NIV or IMV (severity scale ≥5) were all invited to receive the pulmonary function test. The ratio used to select patients not requiring supplemental oxygen (severity scale as 3) and those requiring supplemental oxygen (severity scale as 4) was 1:2. Finally, 516 patients were ascertained as the eligible patients to receive the above tests. A total of 349 survivors had completed pulmonary function tests at the 6-month visit, and they were all invited to perform this test again at 1-year and 2-year visit. The pulmonary function test was conducted using the Master Screen PFT (vyaire Medical GmbH, Hoechberg, Germany) according to American Thoracic Society (ATS) guidelines.[1]

**Sensitivity analyses**

Sensitivity analyses were conducted for identifying monocyte count trajectory from discharge to 2-year after symptom onset and association of monocyte count trajectory and monocyte count at discharge with outcomes. The sensitivity analyses were performed to assess the effect of excluding participants with last monocyte count during hospitalization measured more than 7 days before discharge.

# Results Supplement

**Monocyte count trajectories from discharge to 2-year follow-up**

Of study participants, 84.9% and 15.1% were classified into normal trajectory and high to normal high trajectory, respectively. The high to normal high trajectory included participants with mean value of monocyte count at discharge as 0.68×10^9^/L, reducing to approximately 0.56×10^9^/L at 6 month after symptom onset, and then fluctuating between around 0.51×10^9^/L and 0.50×10^9^/L from 1 year to 2 years after symptom onset. The normal trajectory included participants with mean value of monocyte count fluctuating between around 0.39×10^9^/L and 0.32×10^9^/L from discharge to 2 years after symptom onset.

**Baseline characteristics of study participants**

Participants with higher monocyte count at discharge and at acute phase were more severely ill and more likely to receive antibiotics and intravenous immunoglobulin. Participants with higher level of highest monocyte count at acute phase were more likely to be less educated and admit to ICU, and have longer duration of hospital stay.

**Clinical outcomes and healthcare use of study participants**

Participants with monocyte count ≥0.60×10^9^/L at discharge had significantly higher percentage of smell disorder, rehospitalization after discharge, TLC <80% of predicted, and DLCO <80% of predicted compared to those with monocyte count <0.40×10^9^/L or 0.40- <0.60×10^9^/L at discharge. Participants with highest monocyte count at acute phase ≥0.60×10^9^/L showed higher percentage with TLC <80% of predicted compared to those with highest monocyte count <0.40×10^9^/L or 0.40- <0.60×10^9^/L at acute phase. Although the proportion of participants with DLCO <80% of predicted was numerically higher among those with highest monocyte count at acute phase ≥0.60×10^9^/L compared with those in the other two groups, the difference was not statistically significant.

**Association of highest monocyte count at acute phase with clinical outcomes and healthcare use**

The associations of highest monocyte count at acute phase with clinical outcomes and healthcare use at 2-year follow-up were also analyzed (Figure S4 and Figure S5). The multivariable adjusted ORs (95% CIs) were 4.05 (1.06-15.55) and 4.73 (1.20-18.56) for TLC <80% of predicted in participants with highest monocyte count 0.40-<0.60$\times$10^9^/L and ≥0.60$\times$10^9^/L at acute phase compared with participants with highest monocyte count <0.40×10^9^/L at acute phase, respectively. Compared with participants with highest monocyte count <0.40×10^9^/L at acute phase, the multivariable adjusted ORs (95% CIs) were 1.24 (0.83-1.87) and 1.60 (1.01-2.54) for rehospitalization after discharge in participants with highest monocyte count 0.40-<0.60$\times$10^9^/L and ≥0.60$\times$10^9^/L at acute phase, respectively.

**References**

1. Standardization of Spirometry, 1994 Update. American Thoracic Society. Am J Respir Crit Care Med. 1995;152(3): 1107-36.

# Table S1. Baseline characteristics of hospitalized COVID-19 survivors according to monocyte count at discharge

|  | **Monocyte count at discharge** | | | | | | ***P* value** | |  |
| --- | --- | --- | --- | --- | --- | --- | --- | --- | --- |
|  | **<0.40**$\boldsymbol{\times}$**10^9^/L**  **(n=636)** | | | **0.40-<0.60**$\boldsymbol{\times}$**10^9^/L**  **(n=581)** | | **≥0.60**$\boldsymbol{\times}$**10^9^/L**  **(n=172)** |  |  |  |
| Age, years | 56.0 (47.0-64.0) | | | 57.0 (48.0-65.0) | | 59.0 (49.0-66.0) | 0.08 | |  |
| **Sex** |  | | |  | |  | <0.0001 | |  |
| Male | 280 (44%) | | | 340 (59%) | | 123 (72%) |  | |  |
| Female | 356 (56%) | | | 241 (41%) | | 49 (28%) |  | |  |
| **Education** |  | | |  | |  | 0.18 | |  |
| College or higher | 169/626 (27%) | | | 180/575 (31%) | | 55/170 (32%) |  | |  |
| Middle school or lower | 457/626 (73%) | | | 395/575 (69%) | | 115/170 (68%) |  | |  |
| **Cigarette smoking** |  | | |  | |  | <0.0001 | |  |
| Never-smoker | 555/635 (87%) | | | 480 (83%) | | 116 (67%) |  | |  |
| Current smoker | 35/635 (6%) | | | 43 (7%) | | 25 (15%) |  | |  |
| Former smoker | 45/635 (7%) | | | 58 (10%) | | 31 (18%) |  | |  |
| **BMI, Kg/m^2^** | 24.7 (22.5-27.0) | | | 25.2 (23.1-27.3) | | 25.8 (23.9-27.6) | 0.0013 | |  |
| **Comorbidity** |  | | |  | |  |  | |  |
| Hypertension | 207/635 (33%) | | | 211 (36%) | | 82 (48%) | 0.0012 | |  |
| Diabetes | 94/635 (15%) | | | 84 (14%) | | 22 (13%) | 0.80 | |  |
| Coronary heart diseases | 49 (8%) | | | 64/579 (11%) | | 17 (10%) | 0.13 | |  |
| Cerebrovascular diseases | 39 (6%) | | | 29/580 (5%) | | 11 (6%) | 0.64 | |  |
| Chronic kidney disease | 28 (4%) | | | 24 (4%) | | 10 (6%) | 0.64 | |  |
| Malignancy | 18 (3%) | | | 12 (2%) | | 7 (4%) | 0.36 | |  |
| COPD | 9 (1%) | | | 11 (2%) | | 0 (0%) | 0.06 | |  |
| **Highest seven-category scale during hospital stay** |  | | |  | |  | <0.0001 | |  |
| 3: Not requiring supplemental oxygen | 186 (29%) | | | 124 (21%) | | 31 (18%) |  | |  |
| 4: Requiring supplemental oxygen | 420 (66%) | | | 397 (68%) | | 124 (72%) |  | |  |
| 5: Requiring HFNC or non-IMV, or both | 28 (4%) | | | 57 (10%) | | 14 (8%) |  | |  |
| 6: Requiring ECMO or IMV, or both | 2 (0%) | | | 3 (1%) | | 3 (2%) |  | |  |
| **Treatment received during hospital stay** |  | | |  | |  |  | |  |
| Corticosteroids | 105/633 (17%) | | | 161/580 (28%) | | 66 (38%) | <0.0001 | |  |
| Antivirals | 326/620 (53%) | | | 318/572 (56%) | | 102 (59%) | 0.25 | |  |
| Lopinavir-ritonavir | 74/633 (12%) | | | 77/580 (13%) | | 38 (22%) | 0.0019 | |  |
| Arbidol | 300/633 (47%) | | | 285/580 (49%) | | 82 (48%) | 0.82 | |  |
| Chloroquine phosphate | 1/633 (0%) | | | 3/580 (1%) | | 0 (0%) | 0.62 | |  |
| Hydroxychloroquine | 0/633 (0%) | | | 0/580 (0%) | | 1 (1%) | 0.12 | |  |
| Antibiotics | 476/633 (75%) | | | 447/580 (77%) | | 147 (85%) | 0.017 | |  |
| Thymosin | 99/633 (16%) | | | 101/580 (17%) | | 21 (12%) | 0.25 | |  |
| Intravenous immunoglobulin | 104/633 (16%) | | | 129/580 (22%) | | 42 (24%) | 0.011 | |  |
| Length of hospital stay, days | 14.0 (10.0-19.0) | | | 15.0 (11.0-21.0) | | 14.0 (10.0-19.0) | 0.06 | |  |
| ICU admission | 23 (4%) | | | 31 (5%) | | 9 (5%) | 0.32 | |  |
| Length of ICU stay, days | 7.0 (2.0-14.0) | | | 18.0 (10.0-30.0) | | 37.0 (23.0-55.0) | 0.0004 | |  |
| Vaccination status* | |  |  | |  | | | 0.17 | |
| Unvaccinated | | 108/631 (17%) | 121/579 (21%) | | 30/172 (17%) | | |  | |
| One dose | | 161/631 (26%) | 146/579 (25%) | | 54/172 (31%) | | |  | |
| Two doses | | 304/631 (48%) | 267/579 (46%) | | 68/172 (40%) | | |  | |
| Three and more doses | | 58/631 (9%) | 45/579 (8%) | | 20/172 (12%) | | |  | |
| Time from symptom onset to 2-year follow-up, days | 688.0 (676.0-701.0) | | | 688.0 (676.0-699.0) | | 685.5 (675.0-699.0) | 0.47 | |  |

Data are n (%), n/N(%), or median (IQR). The differing denominators used indicate missing data. NA=not applicable. HFNC=high-flow nasal cannula for oxygen therapy. IMV=invasive mechanical ventilation. ECMO=extracorporeal membrane oxygenation. ICU=intensive care unit.

* Vaccination status at 2-year follow-up visit. COVID-19 survivors in this study were not vaccinated at 6-month and 1-year follow-up.

# Table S2. Baseline characteristics of hospitalized COVID-19 survivors according to highest monocyte count at acute phase

|  | **Highest monocyte count at acute phase** | | | ***P* value** |
| --- | --- | --- | --- | --- |
|  | **<0.40**$\boldsymbol{\times}$**10^9^/L**  **(n=401)** | **0.40-<0.60**$\boldsymbol{\times}$**10^9^/L**  **(n=599)** | **≥0.60**$\boldsymbol{\times}$**10^9^/L**  **(n=389)** |  |
| Age, years | 56.0 (47.0-64.0) | 58.0 (48.0-65.0) | 57.0 (48.0-65.0) | 0.19 |
| **Sex** |  |  |  | <0.0001 |
| Male | 156 (39%) | 314 (52%) | 273 (70%) |  |
| Female | 245 (61%) | 285 (48%) | 116 (30%) |  |
| **Education** |  |  |  | 0.016 |
| College or higher | 111/395 (28%) | 158/591 (27%) | 135/385 (35%) |  |
| Middle school or lower | 284/395 (72%) | 433/591 (73%) | 250/385 (65%) |  |
| **Cigarette smoking** |  |  |  | <0.0001 |
| Never-smoker | 356 (89%) | 502/598 (84%) | 293 (75%) |  |
| Current smoker | 21 (5%) | 43/598 (7%) | 39 (10%) |  |
| Former smoker | 24 (6%) | 53/598 (9%) | 57 (15%) |  |
| **BMI, Kg/m^2^** | 24.3 (21.8-26.5) | 25.0 (23.0-27.3) | 25.8 (23.8-27.8) | <0.0001 |
| **Comorbidity** |  |  |  |  |
| Hypertension | 112 (28%) | 220/598 (37%) | 168 (43%) | <0.0001 |
| Diabetes | 60/400 (15%) | 85 (14%) | 55 (14%) | 0.92 |
| Coronary heart diseases | 33 (8%) | 57/598 (10%) | 40/388 (10%) | 0.60 |
| Cerebrovascular diseases | 22 (5%) | 32/598 (5%) | 25 (6%) | 0.76 |
| Chronic kidney disease | 19 (5%) | 25 (4%) | 18 (5%) | 0.90 |
| Malignancy | 10 (2%) | 17 (3%) | 10 (3%) | 0.94 |
| COPD | 5 (1%) | 11 (2%) | 4 (1%) | 0.54 |
| **Highest seven-category scale during hospital stay** |  |  |  | <0.0001 |
| 3: Not requiring supplemental oxygen | 144 (36%) | 140 (23%) | 57 (15%) |  |
| 4: Requiring supplemental oxygen | 251 (63%) | 432 (72%) | 258 (66%) |  |
| 5: Requiring HFNC or non-IMV, or both | 6 (1%) | 27 (5%) | 66 (17%) |  |
| 6: Requiring ECMO or IMV, or both | 0 (0%) | 0 (0%) | 8 (2%) |  |
| **Treatment received during hospital stay** |  |  |  |  |
| Corticosteroids | 42/399 (11%) | 129/598 (22%) | 161/388 (41%) | <0.0001 |
| Antivirals | 201/392 (51%) | 318/588 (54%) | 227/384 (59%) | 0.08 |
| Lopinavir-ritonavir | 44/399 (11%) | 64/598 (11%) | 81/388 (21%) | <0.0001 |
| Arbidol | 183/399 (46%) | 295/598 (49%) | 189/388 (49%) | 0.54 |
| Chloroquine phosphate | 0/399 (0%) | 1/598 (0%) | 3/388 (1%) | 0.09 |
| Hydroxychloroquine | 0/399 (0%) | 0/598 (0%) | 1/388 (0%) | 0.28 |
| Antibiotics | 275/399 (69%) | 455/598 (76%) | 340/388 (88%) | <0.0001 |
| Thymosin | 59/399 (15%) | 107/598 (18%) | 55/388 (14%) | 0.22 |
| Intravenous immunoglobulin | 46/399 (12%) | 114/598 (19%) | 115/388 (30%) | <0.0001 |
| Length of hospital stay, days | 12.0 (8.0-16.0) | 14.0 (10.0-19.0) | 17.0 (12.0-29.0) | <0.0001 |
| ICU admission | 7 (2%) | 17 (3%) | 39 (10%) | <0.0001 |
| Length of ICU stay, days | 3.0 (2.0-7.0) | 8.0 (5.0-14.0) | 21.0 (10.0-40.0) | 0.0001 |
| Vaccination status* |  |  |  | 0.40 |
| Unvaccinated | 61/398 (15%) | 116/595 (19%) | 82/389 (21%) |  |
| One dose | 103/398 (26%) | 152/595 (26%) | 106/389 (27%) |  |
| Two doses | 195/398 (49%) | 277/595 (47%) | 167/389 (43%) |  |
| Three and more doses | 39/398 (10%) | 50/595 (8%) | 34/389 (9%) |  |

Data are n (%), n/N(%), or median (IQR). The differing denominators used indicate missing data. NA=not applicable. HFNC=high-flow nasal cannula for oxygen therapy. IMV=invasive mechanical ventilation. ECMO=extracorporeal membrane oxygenation. ICU=intensive care unit.

* Vaccination status at 2-year follow-up visit. COVID-19 survivors in this study were not vaccinated at 6-month and 1-year follow-up.

# Table S3. Clinical outcomes and healthcare use of hospitalized COVID-19 survivors at 2-year follow-up visits according to monocyte count at discharge

|  | **Monocyte count at discharge** | | | ***P* value** |
| --- | --- | --- | --- | --- |
|  | **<0.40**$\boldsymbol{\times}$**10^9^/L**  **(n=636)** | **0.40-<0.60**$\boldsymbol{\times}$**10^9^/L**  **(n=581)** | **≥0.60**$\boldsymbol{\times}$**10^9^/L**  **(n=172)** |  |
| **Core symptom*** |  |  |  |  |
| Fatigue or muscle weakness | 192/634 (30%) | 173/580 (30%) | 48/172 (28%) | 0.83 |
| Smell disorder | 26/634 (4%) | 29/580 (5%) | 17/172 (10%) | 0.0097 |
| Taste disorder | 22/634 (3%) | 18/580 (3%) | 4/172 (2%) | 0.74 |
| Dyspnoea (mMRC≥1) | 88/635 (14%) | 86/580 (15%) | 28/172 (16%) | 0.71 |
| **Distance walked in 6 min, m** | 511.0 (458.0-555.0) | 510.0 (455.0-558.0) | 525.0 (470.0-575.0) | 0.21 |
| Percentage of predicted value† | 93.6 (84.9-102.5) | 94.1 (84.5-104.5) | 94.4 (85.5-106.8) | 0.37 |
| Less than LLN‡ | 35/481 (7%) | 38/447 (9%) | 15/141 (11%) | 0.43 |
| **EQ-5D-5L questionnaire** |  |  |  |  |
| Pain or discomfort | 148/635 (23%) | 136/580 (23%) | 41 (24%) | 0.99 |
| Anxiety or depression | 90/635 (14%) | 77/580 (13%) | 18 (10%) | 0.45 |
| Mobility problem | 21/635 (3%) | 24/580 (4%) | 5 (3%) | 0.64 |
| Usual activity problem | 18/635 (3%) | 21/580 (4%) | 4 (2%) | 0.60 |
| Personal care problem | 5/635 (1%) | 11/580 (2%) | 3 (2%) | 0.21 |
| EuroQol VAS score | 80.0 (70.0-90.0) | 80.0 (70.0-90.0) | 80.0 (70.0-90.0) | 0.33 |
| **Healthcare use after discharge** |  |  |  |  |
| Outpatient clinic visit | 122/635 (19%) | 116/577 (20%) | 36 (21%) | 0.86 |
| Rehospitalization | 77/635 (12%) | 76/577 (13%) | 34 (20%) | 0.032 |
| Emergency department visit | 6/635 (1%) | 1/577 (0%) | 2 (1%) | 0.12 |
| **Lung function** |  |  |  |  |
| FEV_1_, L | 2.7 (2.3-3.3) | 2.9 (2.4-3.6) | 3.1 (2.5-3.6) | 0.13 |
| FVC, L | 3.5 (3.0-4.1) | 3.7 (3.1-4.4) | 3.8 (3.3-4.3) | 0.18 |
| TLC, L | 4.9 (4.2-5.6) | 5.2 (4.5-5.7) | 5.0 (4.6-5.7) | 0.23 |
| DLCO, mmol/min/kPa | 6.7 (5.5-7.9) | 6.9 (6.0-8.3) | 6.4 (6.0-7.4) | 0.08 |
| FEV_1_ <80%, % of predicted | 6/102 (6%) | 7/124 (6%) | 0/34 (0%) | 0.15 |
| FVC <80%, % of predicted | 5/102 (5%) | 4/124 (3%) | 0/34 (0%) | 0.23 |
| TLC <80%, % of predicted | 14/101 (14%) | 26/124 (21%) | 16/34 (47%) | 0.0002 |
| DLCO <80%, % of predicted | 44/102 (43%) | 49/124 (40%) | 22/34 (65%) | 0.031 |

Data are median (IQR), n(%) or n/N (%). The differing denominators used indicate missing data. DLCO=diffusion capacity for carbon monoxide. EQ-5D-5L=EuroQol five-dimension five-level questionnaire. EQ-VAS=EuroQol Visual Analogue Scale. FEV_1_=forced expiratory volume in 1s. FVC=forced vital capacity. mMRC= modified British Medical Research Council. TLC=total lung capacity. LLN=lower limit of normal range.

*Core symptoms were identified as symptoms more specifically related to long COVID according to findings of Mizrahi and Ballering [26,27].

†Predicted values were calculated according to the method of Enright and Sherrill.

‡The lower limit of the normal range was calculated by subtracting 153 m from the predicted value for men or by subtracting 139 m for women.

# Table S4. Clinical outcomes and healthcare use of hospitalized COVID-19 survivors at 2-year follow-up visits according to highest monocyte count at acute phase

|  | **Highest monocyte count at acute phase** | | | ***P* value** |
| --- | --- | --- | --- | --- |
|  | **<0.40**$\boldsymbol{\times}$**10^9^/L**  **(n=401)** | **0.40-<0.60**$\boldsymbol{\times}$**10^9^/L**  **(n=599)** | **≥0.60**$\boldsymbol{\times}$**10^9^/L**  **(n=389)** |  |
| **Core symptom*** |  |  |  |  |
| Fatigue or muscle weakness | 122/401 (30%) | 169/596 (28%) | 122/389 (31%) | 0.57 |
| Smell disorder | 18/401 (4%) | 29/596 (5%) | 25/389 (6%) | 0.42 |
| Taste disorder | 14/401 (3%) | 17/596 (3%) | 13/389 (3%) | 0.83 |
| Dyspnoea (mMRC≥1) | 57/401 (14%) | 83/597 (14%) | 62/389 (16%) | 0.66 |
| **Distance walked in 6 min, m** | 510.0 (458.0-555.0) | 510.0 (455.0-555.0) | 524.0 (466.0-570.0) | 0.12 |
| Percentage of predicted value† | 93.9 (85.3-102.7) | 93.5 (84.6-104.1) | 94.7 (84.4-106.0) | 0.56 |
| Less than LLN‡ | 25/300 (8%) | 34/462 (7%) | 29/307 (9%) | 0.59 |
| **EQ-5D-5L questionnaire** |  |  |  |  |
| Pain or discomfort | 94 (23%) | 153/597 (26%) | 78 (20%) | 0.13 |
| Anxiety or depression | 57 (14%) | 84/597 (14%) | 44 (11%) | 0.38 |
| Mobility problem | 12 (3%) | 23/597 (4%) | 15 (4%) | 0.74 |
| Usual activity problem | 12 (3%) | 21/597 (4%) | 10 (3%) | 0.70 |
| Personal care problem | 5 (1%) | 7/597 (1%) | 7 (2%) | 0.69 |
| EuroQol VAS score | 80.0 (70.0-90.0) | 80.0 (70.0-90.0) | 80.0 (70.0-90.0) | 0.57 |
| **Healthcare use after discharge** |  |  |  |  |
| Outpatient clinic visit | 85 (21%) | 115/595 (19%) | 74/388 (19%) | 0.70 |
| Rehospitalization | 46 (11%) | 78/595 (13%) | 63/388 (16%) | 0.14 |
| Emergency department visit | 3 (1%) | 4/595 (1%) | 2/388 (1%) | 0.91 |
| **Lung function** |  |  |  |  |
| FEV_1_, L | 2.7 (2.4-3.4) | 2.7 (2.3-3.3) | 3.1 (2.5-3.6) | 0.09 |
| FVC, L | 3.5 (3.1-4.3) | 3.5 (3.0-4.1) | 3.9 (3.2-4.4) | 0.21 |
| TLC, L | 5.0 (4.4-5.8) | 4.9 (4.4-5.4) | 5.1 (4.3-5.7) | 0.68 |
| DLCO, mmol/min/kPa | 6.9 (5.8-8.0) | 6.7 (5.8-8.0) | 6.8 (5.6-8.2) | 0.96 |
| FEV_1_ <80%, % of predicted | 2/51 (4%) | 5/99 (5%) | 6/110 (5%) | 0.91 |
| FVC <80%, % of predicted | 1/51 (2%) | 3/99 (3%) | 5/110 (5%) | 0.66 |
| TLC <80%, % of predicted | 3/51 (6%) | 19/98 (19%) | 34/110 (31%) | 0.0013 |
| DLCO <80%, % of predicted | 22/51 (43%) | 37/99 (37%) | 56/110 (51%) | 0.14 |

Data are median (IQR), n(%) or n/N (%). The differing denominators used indicate missing data. DLCO=diffusion capacity for carbon monoxide. EQ-5D-5L=EuroQol five-dimension five-level questionnaire. EQ-VAS=EuroQol Visual Analogue Scale. FEV_1_=forced expiratory volume in 1s. FVC=forced vital capacity. mMRC= modified British Medical Research Council. TLC=total lung capacity. LLN=lower limit of normal range.

*Core symptoms were identified as symptoms more specifically related to long COVID according to findings of Mizrahi and Ballering [26,27].

†Predicted values were calculated according to the method of Enright and Sherrill.

‡The lower limit of the normal range was calculated by subtracting 153 m from the predicted value for men or by subtracting 139 m for women.

# Table S5. Association of monocyte count trajectories with outcomes with further adjustment for vaccination status at 2-year follow-up

|  | OR or ß (95% CI) | *P* value |
| --- | --- | --- |
| Fatigue or muscle weakness |  |  |
| Normal | ref |  |
| High to normal high | 0.94 (0.66, 1.32) | 0.71 |
| Smell disorder |  |  |
| Normal | ref |  |
| High to normal high | 2.59 (1.47, 4.55) | 0.0010 |
| Taste disorder |  |  |
| Normal | ref |  |
| High to normal high | 0.92 (0.37, 2.32) | 0.86 |
| Dyspnoea |  |  |
| Normal | ref |  |
| High to normal high | 1.00 (0.64, 1.55) | 0.99 |
| Distance walked in 6 min, m |  |  |
| Normal | ref |  |
| High to normal high | -1.41 (-14.9,12.04) | 0.84 |
| Percentage of predicted 6MWD |  |  |
| Normal | ref |  |
| High to normal high | 0.71 (-2.02, 3.44) | 0.61 |
| 6MWD less than LLN |  |  |
| Normal | ref |  |
| High to normal high | 2.24 (1.25, 4.01) | 0.0066 |
| FEV_1_<80% of predicted value |  |  |
| Normal | ref |  |
| High to normal high | 0.53 (0.09, 3.10) | 0.48 |
| FVC<80% of predicted value |  |  |
| Normal |  |  |
| High to normal high | NA | NA |
| TLC<80% of predicted value |  |  |
| Normal | ref |  |
| High to normal high | 2.70 (1.16, 6.29) | 0.021 |
| DLCO <80% of predicted value |  |  |
| Normal | ref |  |
| High to normal high | 1.54 (0.72, 3.29) | 0.26 |
| Pain or discomfort |  |  |
| Normal | ref |  |
| High to normal high | 1.29 (0.90, 1.86) | 0.17 |
| Anxiety or depression |  |  |
| Normal | ref |  |
| High to normal high | 0.90 (0.55, 1.49) | 0.69 |
| Mobility problem |  |  |
| Normal | ref |  |
| High to normal high | 1.77 (0.85, 3.69) | 0.13 |
| Usual activity problem |  |  |
| Normal | ref |  |
| High to normal high | 2.03 (0.95, 4.35) | 0.07 |
| Personal care problem |  |  |
| Normal | ref |  |
| High to normal high | 3.39 (1.14,10.06) | 0.028 |
| Outpatient clinic visit |  |  |
| Normal | ref |  |
| High to normal high | 1.07 (0.72, 1.57) | 0.74 |
| Rehospitalization |  |  |
| Normal | ref |  |
| High to normal high | 1.60 (1.05, 2.44) | 0.027 |
| Emergency department visit |  |  |
| Normal | ref |  |
| High to normal high | 1.77 (0.30,10.45) | 0.53 |

# Table S6. Laboratory tests of hospitalized COVID-19 survivors according to monocyte count trajectories

|  | **Total**  **(n=1389)** | **Monocyte count trajectories** | | ***P* value** |
| --- | --- | --- | --- | --- |
|  |  | **Normal**  **(n=1179)** | **High to**  **normal high**  **(n=210)** |  |
| **At discharge** |  |  |  |  |
| Lymphocyte count, × 10^9^ per L | 1.5 (1.2-1.9) | 1.4 (1.1-1.8) | 1.8 (1.4-2.3) | <0.0001 |
| Neutrophil count, × 10^9^ per L | 3.4 (2.6-4.4) | 3.2 (2.5-4.1) | 4.5 (3.8-6.2) | <0.0001 |
| **6-month follow-up** |  |  |  |  |
| Lymphocyte count, × 10^9^ per L | 1.9 (1.5-2.4) | 1.9 (1.5-2.3) | 2.1 (1.6-3.0) | <0.0001 |
| Neutrophil count, × 10^9^ per L | 3.6 (2.9-4.4) | 3.4 (2.8-4.2) | 4.2 (3.6-5.7) | <0.0001 |
| Eosinophils count, × 10^9^ per L | 0.10 (0.06-0.16) | 0.10 (0.06-0.16) | 0.14 (0.08-0.22) | 0.0010 |
| **1-year follow-up** |  |  |  |  |
| Lymphocyte count, × 10^9^ per L | 2.0 (1.6-2.4) | 1.9 (1.5-2.3) | 2.3 (1.9-2.8) | <0.0001 |
| Neutrophil count, × 10^9^ per L | 3.5 (2.9-4.5) | 3.4 (2.8-4.2) | 4.5 (3.7-5.3) | <0.0001 |
| Eosinophils count, × 10^9^ per L | 0.11 (0.06-0.17) | 0.10 (0.06-0.16) | 0.15 (0.10-0.23) | <0.0001 |
| **2-year follow-up** |  |  |  |  |
| Lymphocyte count, × 10^9^ per L | 1.8 (1.5-2.3) | 1.8 (1.4-2.2) | 2.2 (1.7-2.7) | <0.0001 |
| Neutrophil count, × 10^9^ per L | 3.6 (2.9-4.4) | 3.4 (2.8-4.2) | 4.5 (3.7-5.4) | <0.0001 |
| Eosinophils count, × 10^9^ per L | 0.10 (0.06-0.17) | 0.10 (0.06-0.16) | 0.15 (0.09-0.23) | <0.0001 |

# Figure S1. Sensitivity analysis for trajectories of monocyte count from discharge to 2 years after symptom onset among hospitalized COVID-19 survivors


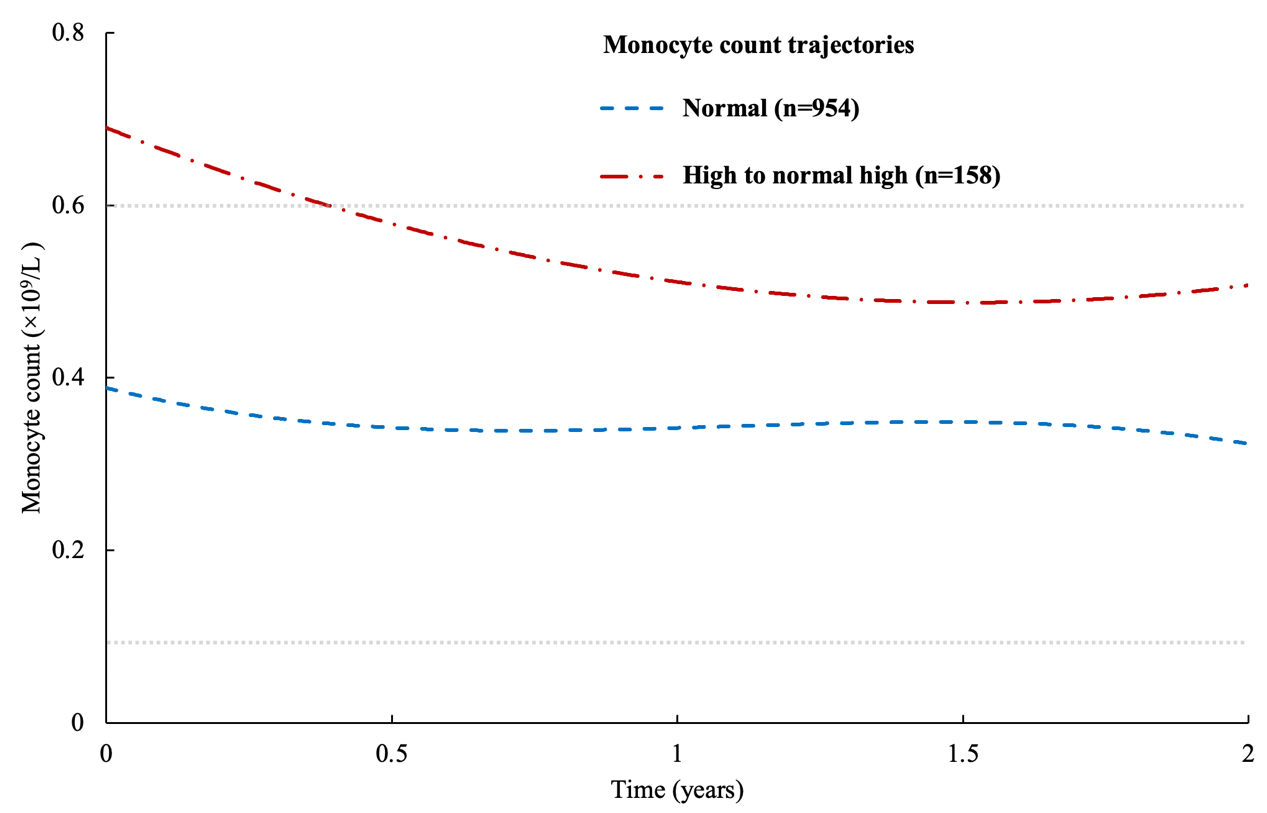


The dashed horizontal lines indicate the normal reference values of monocyte count.

# Figure S2. Sensitivity analysis for association of monocyte count trajectories and monocyte count at discharge with core symptom (a), distance walked in 6 min (b) and lung function (c) at 2-year follow-up

**
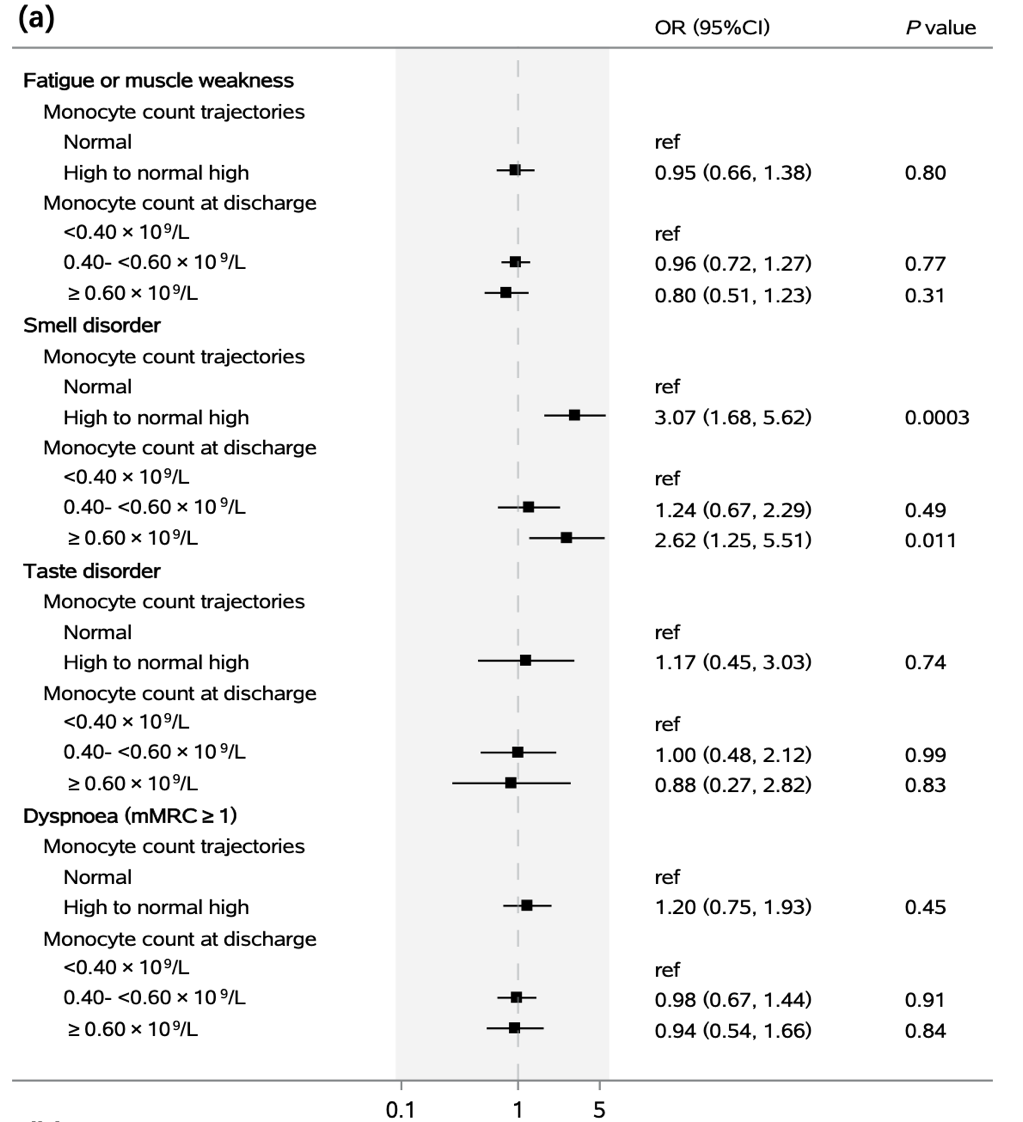
**

**
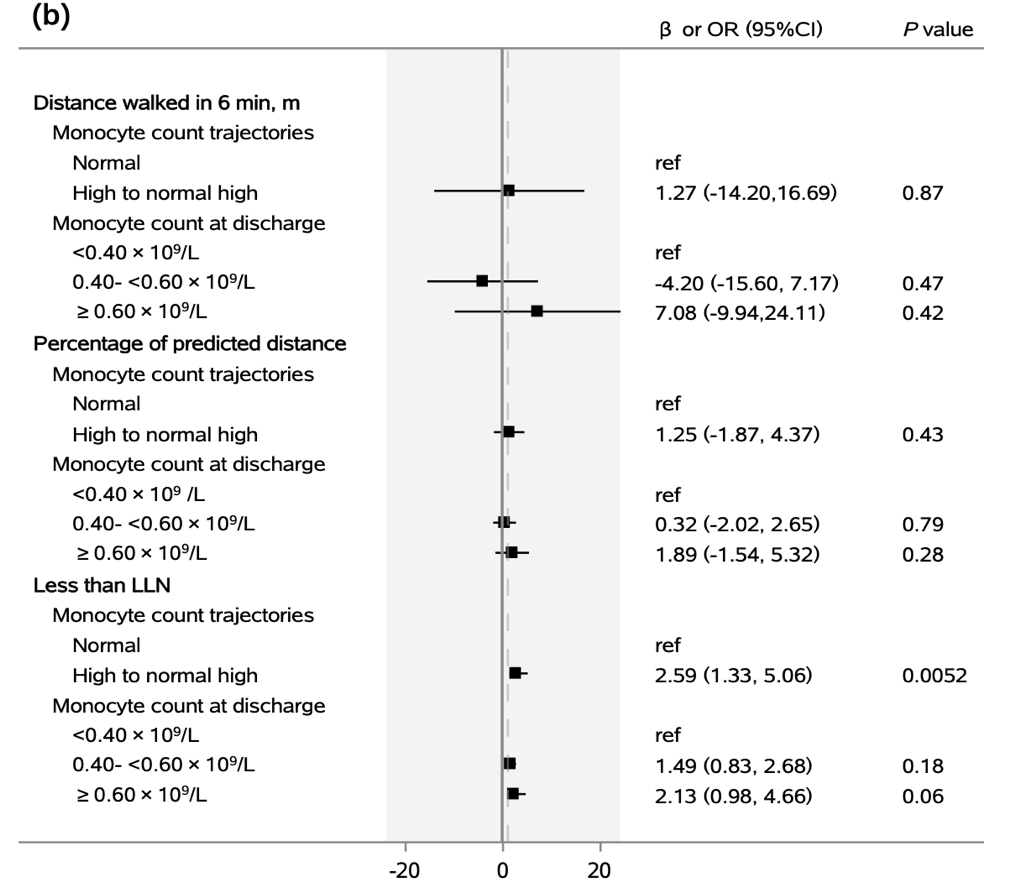
**


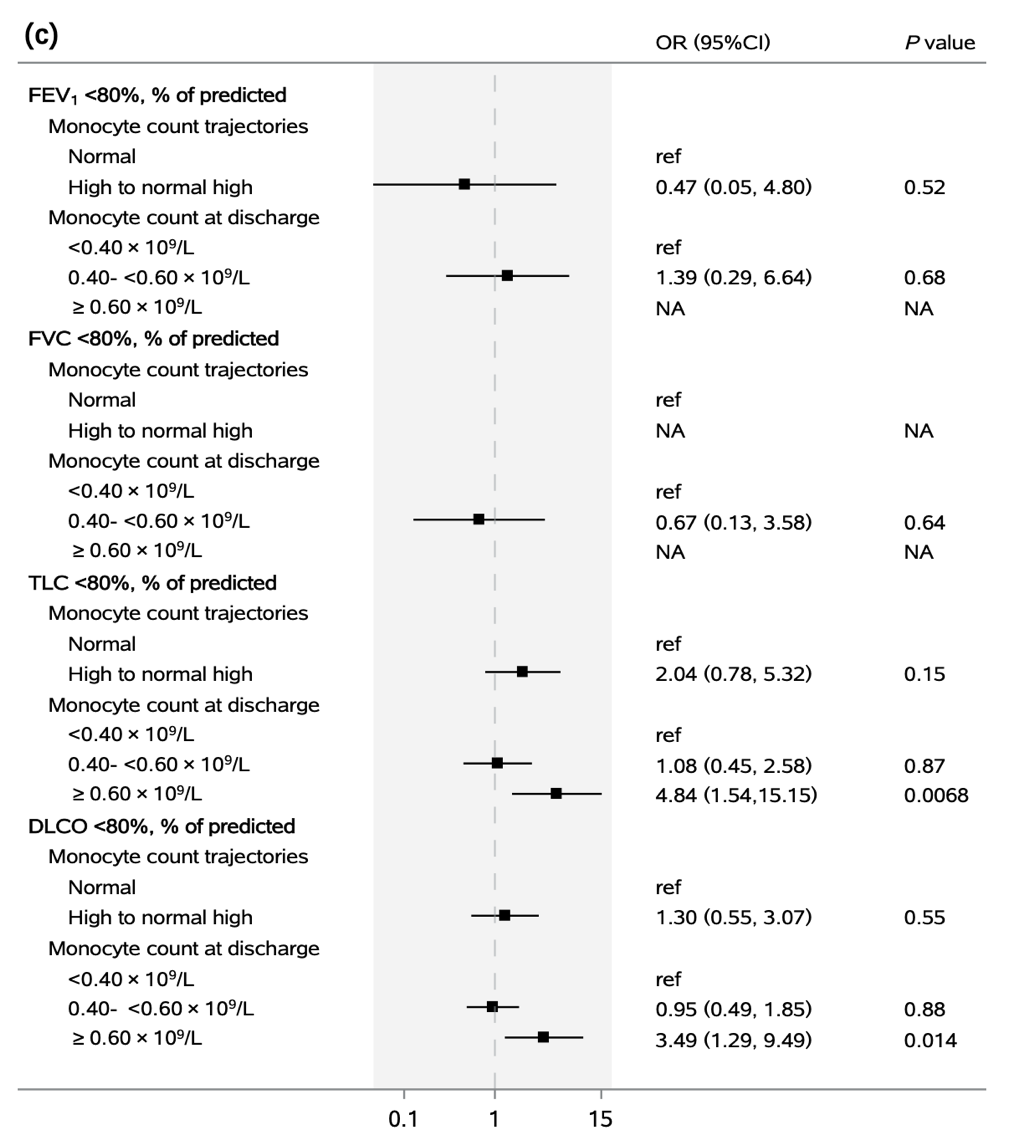


# Figure S3. Sensitivity analysis for association of monocyte count trajectories and monocyte count at discharge with health-related quality of life (a) and healthcare use after discharge (b) at 2-year follow-up


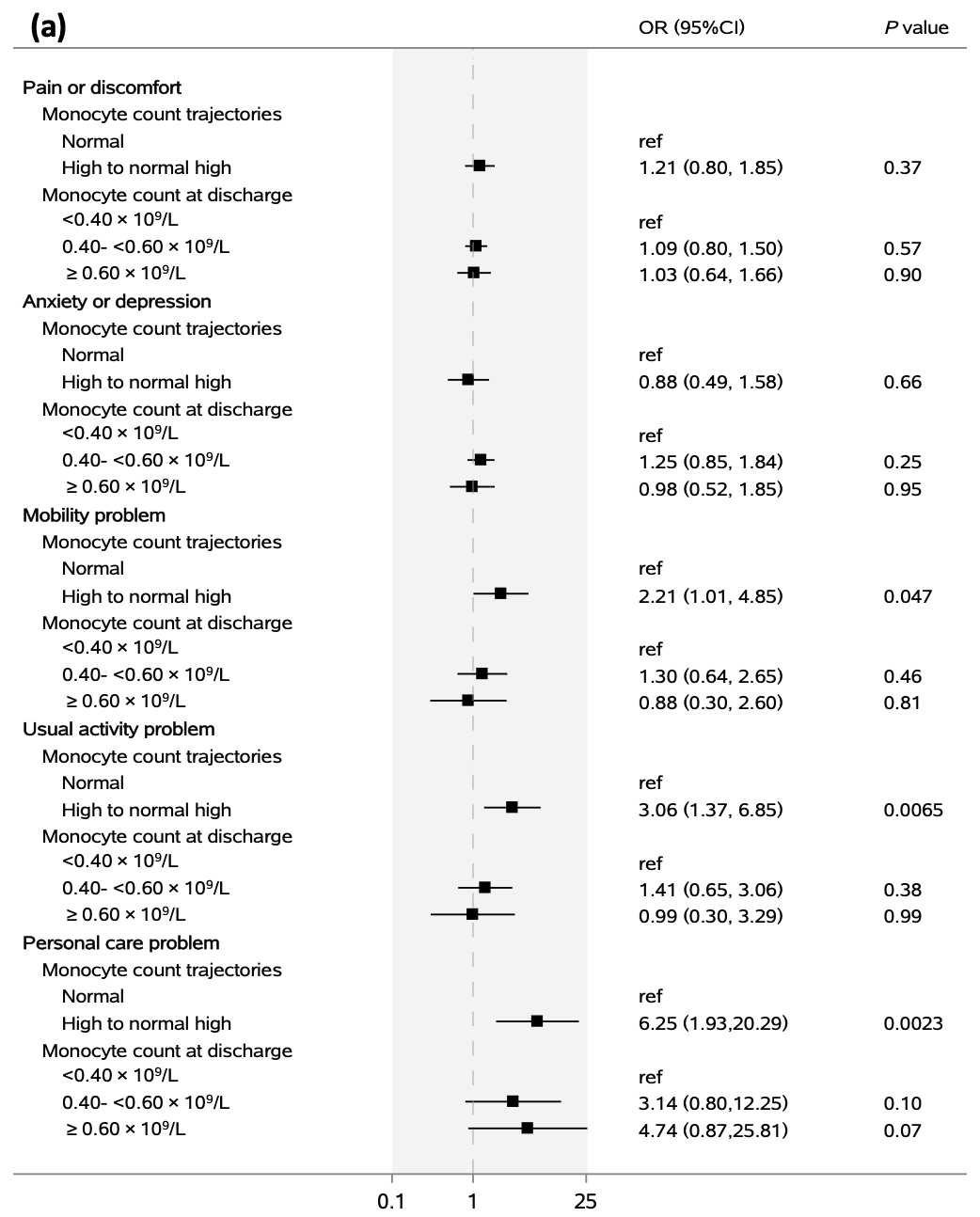


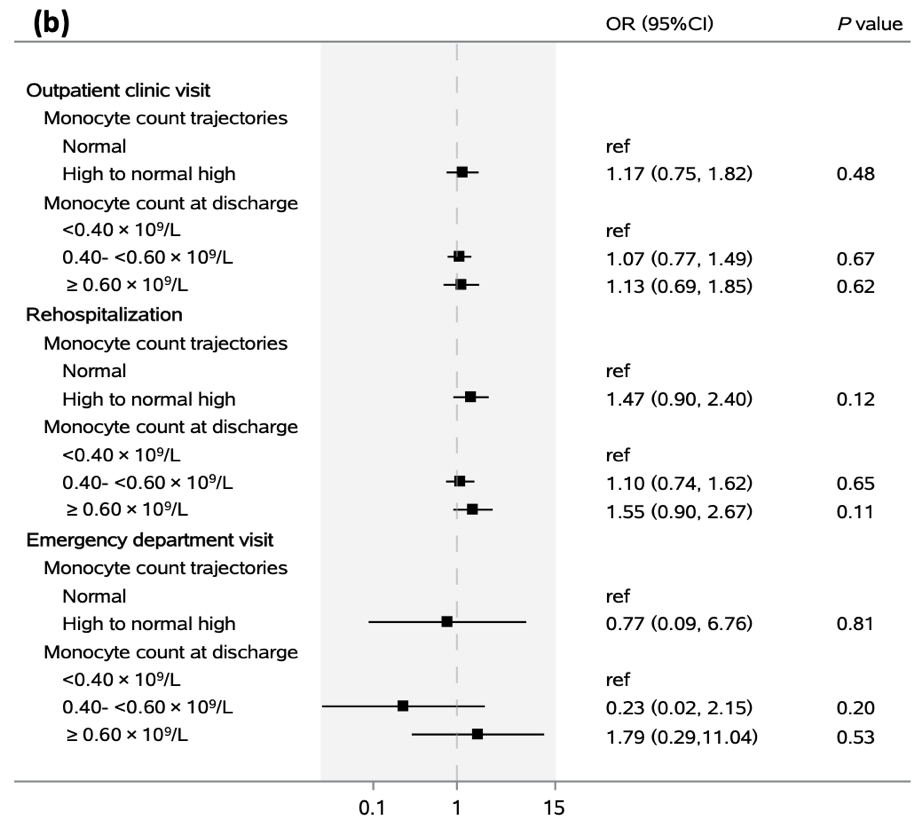


# Figure S4. Association of highest monocyte count at acute phase with core symptom (a), distance walked in 6 min (b) and lung function (c) at 2-year follow-up

**
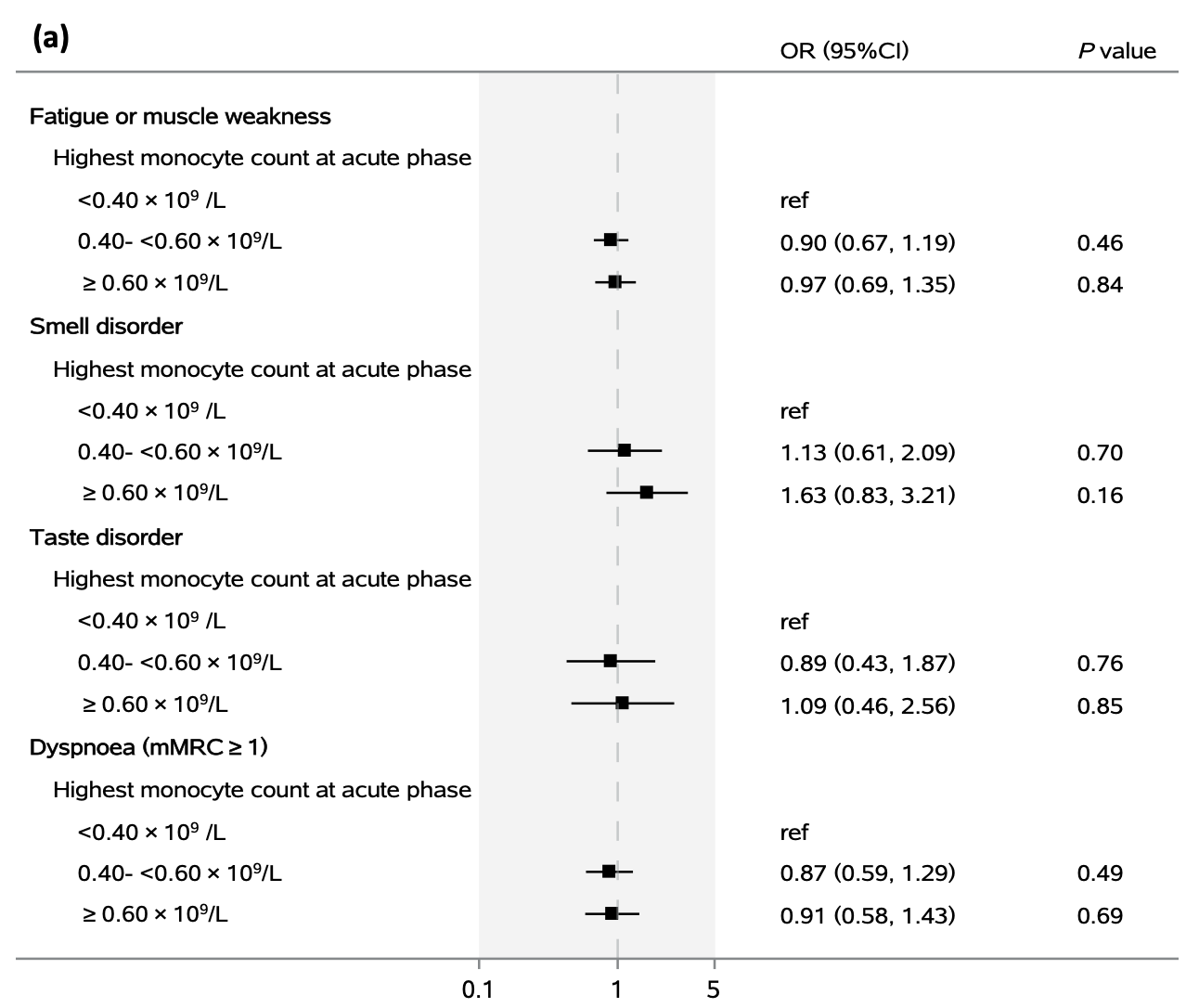
**

**
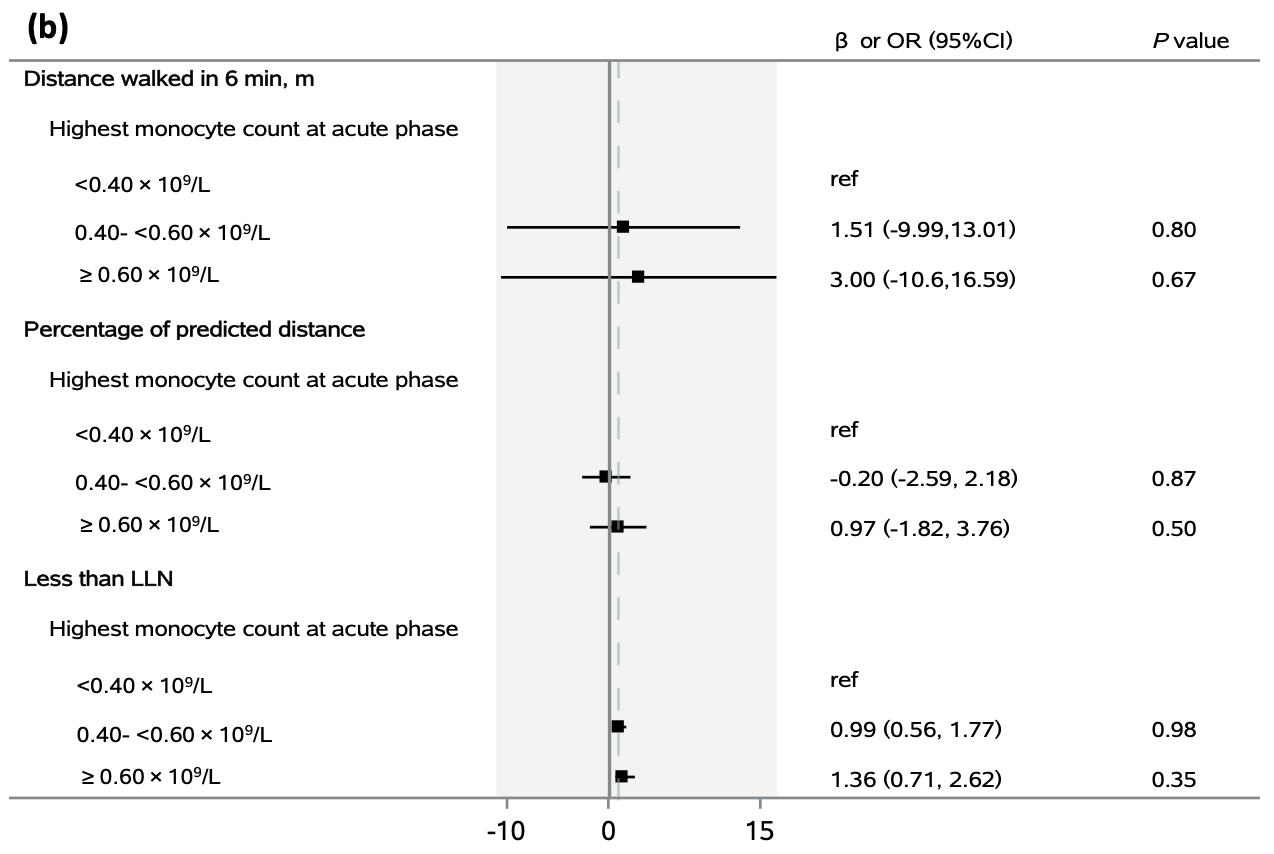
**


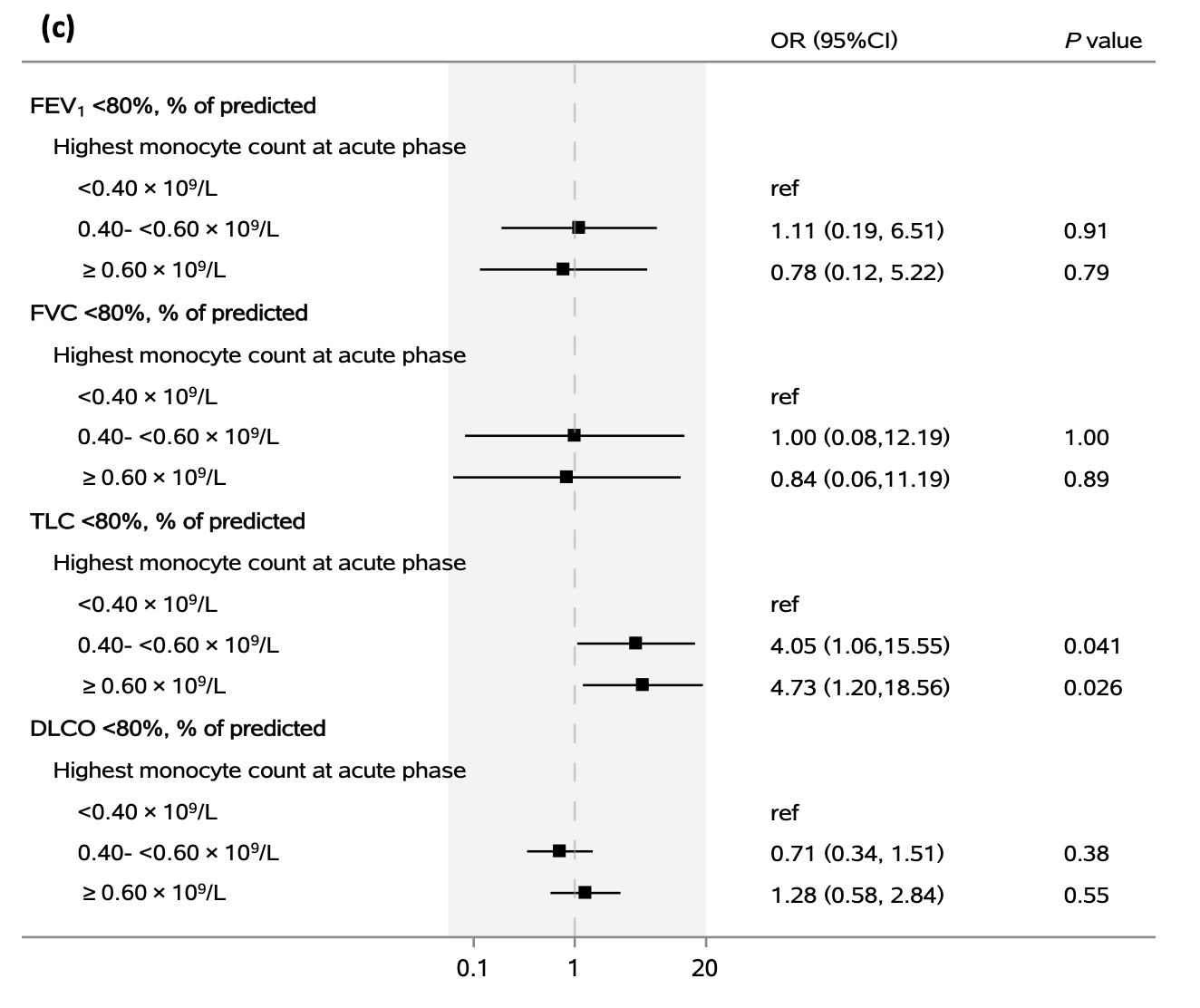


# Figure S5. Association of highest monocyte count at acute phase with health-related quality of life (a) and healthcare use after discharge (b) at 2-year follow-up


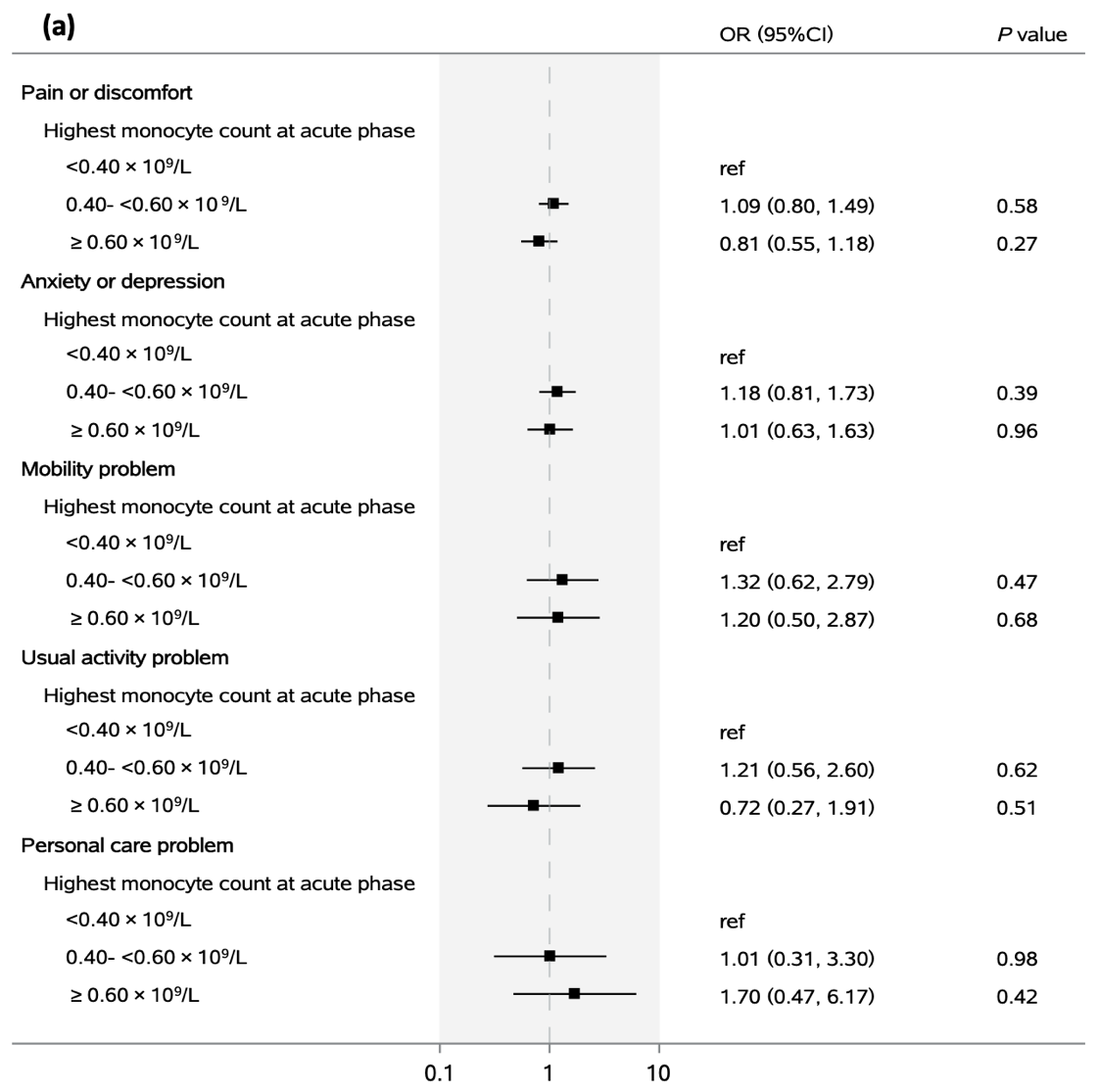


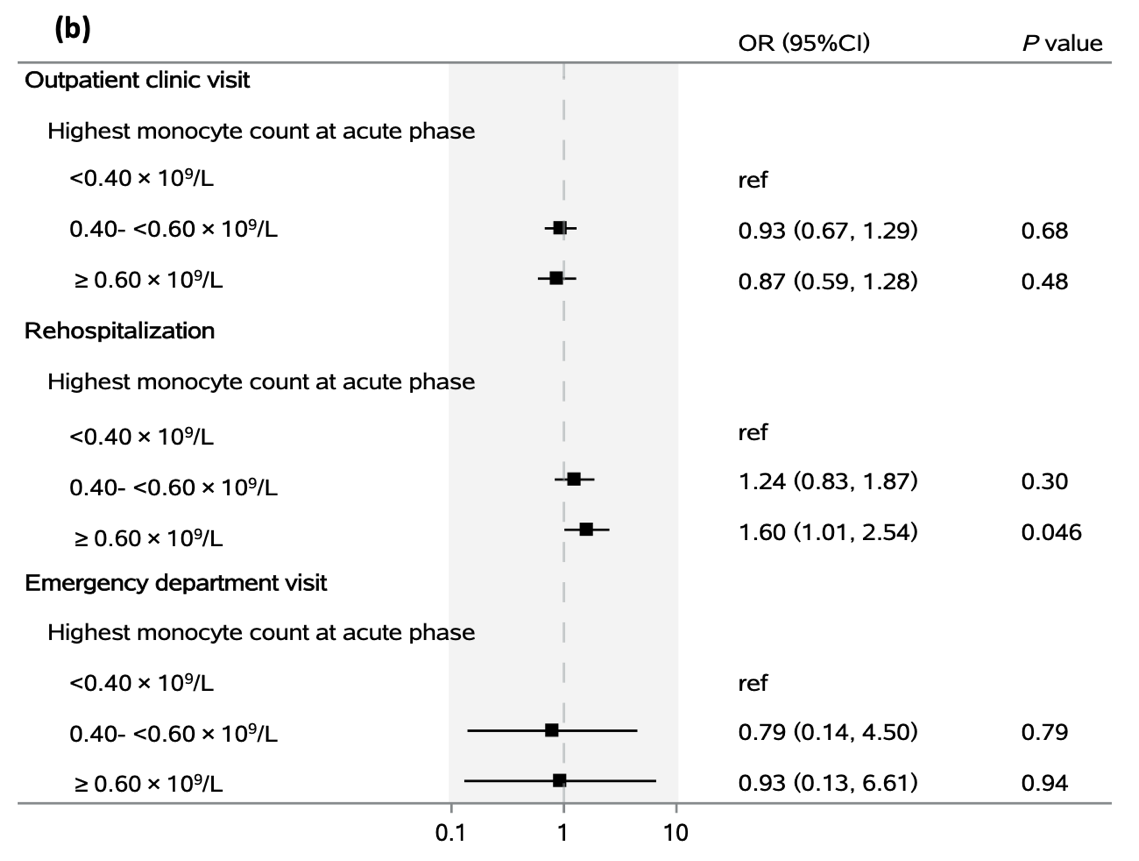

Supplement: Supplementary file 1 — Table S1. Baseline characteristics of hospitalized COVID‐19 survivors according to monocyte count at discharge. Table S2. Baseline characteristics of hospitalized COVID‐19 survivors according to highest monocyte count at acute phase. Table S3. Clinical outcomes and healthcare use of hospitalized COVID‐19 survivors at 2‐year follow‐up visits according to monocyte count at discharge. Table S4. Clinical outcomes and healthcare use of hospitalized COVID‐19 survivors at 2‐year follow‐up visits according to highest monocyte count at acute phase. Table S5. Association of monocyte count trajectories with outcomes with further adjustment for vaccination status at 2‐year follow‐up. Table S6. Laboratory tests of hospitalized COVID‐19 survivors according to monocyte count trajectories. Figure S1. Sensitivity analysis for trajectories of monocyte count from discharge to 2 years after symptom onset among hospitalized COVID‐19 survivors. Figure S2. Sensitivity analysis for association of monocyte count trajectories and monocyte count at discharge with core symptom (a), distance walked in 6 min (b) and lung function (c) at 2‐year follow‐up. Figure S3. Sensitivity analysis for association of monocyte count trajectories and monocyte count at discharge with health‐related quality of life (a) and healthcare use after discharge (b) at 2‐year follow‐up. Figure S4. Association of highest monocyte count at acute phase with core symptom (a), distance walked in 6 min (b) and lung function (c) at 2‐year follow‐up. Figure S5. Association of highest monocyte count at acute phase with health‐related quality of life (a) and healthcare use after discharge (b) at 2‐year follow‐up. [file IRV-18-e13263-s001.docx]
